# Supplementary material for: Stepwise taming of triplet excitons via multiple confinements in intrinsic polymers for long-lived room-temperature phosphorescence
Source: Nat Commun. 2023 Nov 9;14:7252. doi: 10.1038/s41467-023-43133-1 (PMC10636106; doi:10.1038/s41467-023-43133-1)
Supplement: Supplementary file 1 — Supplementary Information [file 41467_2023_43133_MOESM1_ESM.pdf]

Supplementary Information

Stepwise taming of triplet excitons via multiple confinements in intrinsic polymers for long-lived room-temperature phosphorescence

Gao et al.

Supplementary Methods

Materials

The following reagents used in experiments including 2,2′-azobis(2-methylpropionitrile) (AIBN, 98%, recrystallized from 95% ethanol before use), boric acid (BA, 99%), 1-vinylnaphthalene (1VN, 95%), 2-vinylnaphthalene (2VN, 97%), 9-vinylanthracene (9VA, 98%), vinylimidazole (MZ, 98%), *N*-vinylphthalimide (NVP, 98%), and vinyl acetate (VAc, 98%) were purchased from Energy Chemical. Polyvinylalcohol (PVA, approx. Mw 47000, 98.0~99.0 % hydrolyzed) and poly(vinyl acetate) (PVAc, approx. Mw 50000) were purchased from Macklin Inc. 95% Ethanol (AR), hydrochloric acid (AR), methanol (AR), and sodium hydroxide (NaOH, 97%) were purchased from Chuan Dong Chemical, or Chron Chemicals. Deionized water was used in the experiments. Unless otherwise stated, all the chemicals were used directly without further purification.

Synthetic routes

The synthetic routes of the systems are shown below.

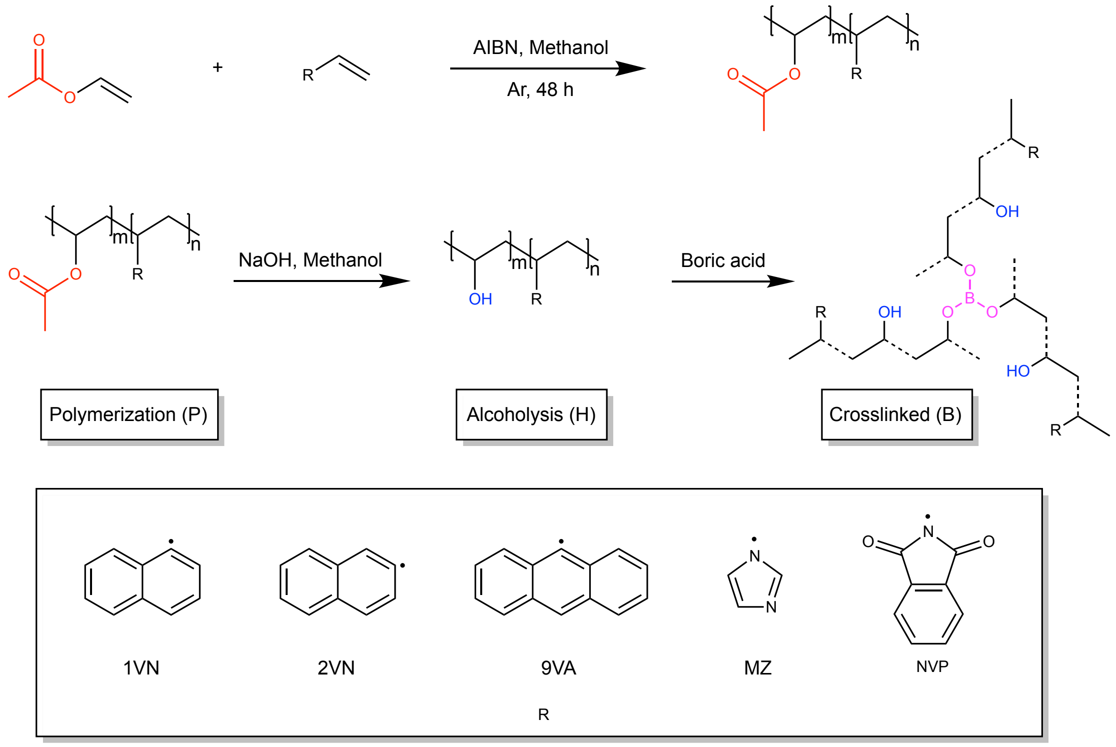

Supplementary Figure 1. Synthetic routes of the systems in this work.

Kinetic calculations and photophysical rate constants

Based on the measured fluorescence and phosphorescence quantum yield efficiency and emission lifetime, the radiative and nonradiative decay rates and intersystem crossing (ISC) rate can be calculated according to standard photophysical dynamic equations (1-5).[1-3] It is considered that the internal conversion rate constant (k_{ic}) is approximately negligible when the energy gap (ΔE) between S1 and S0 for material is larger than 2.17 eV (λ< 571 nm) according to the formula ΔE = 1240/λ.[4-6] k_r^F and k_{nr}^F are the radiative and nonradiative rate constants of fluorescence. k_r^P and k_{nr}^P are the radiative and nonradiative rate constants of room-temperature phosphorescence, Φ_F and Φ_P are the quantum yield efficiency of fluorescence and phosphorescence, τ_F and τ_P are the emission lifetime of fluorescence and phosphorescence respectively. The relationship between them is defined by the following formula (1-5).

$$k_r^F = \frac{\Phi_F}{\tau_F} \quad (1)$$

$$k_{nr}^F = \frac{1-\Phi_F-\Phi_P}{\tau_F} \quad (2)$$

$$k_{isc} = \frac{\Phi_P}{\tau_F} \quad (3)$$

$$k_r^P = \frac{\Phi_P}{\tau_P} \quad (4)$$

$$k_{nr}^P = \frac{1-\Phi_P}{\tau_P} \quad (5)$$

Theoretical calculation details

The structure of polymerization (P), alcoholysis (H), and cross-linked (B) from 1VN, 2VN, 9VA, MZ, and NVP were optimized with dispersion-corrected density functional theory (DFT-D3) at the PBE0-D3/def2-SVP level, SOC, and the energy of S1 and T1 was calculated at PBE0/def2-SVP with the time-dependent density functional theory (TD-DFT) method using the ORCA 5.0 program[7]. The electrostatic potential (ESP) [8,9] values and IRI were calculated by the Multiwfn 3.8(dev) program B3LYPD3 GCP(DFT/TZ)def2-TZVP[10]. The distributions of ESP and IRI pictures were obtained from VMD 1.9.3 program[11]. Simplified models of the

polymeric systems are shown as follows.

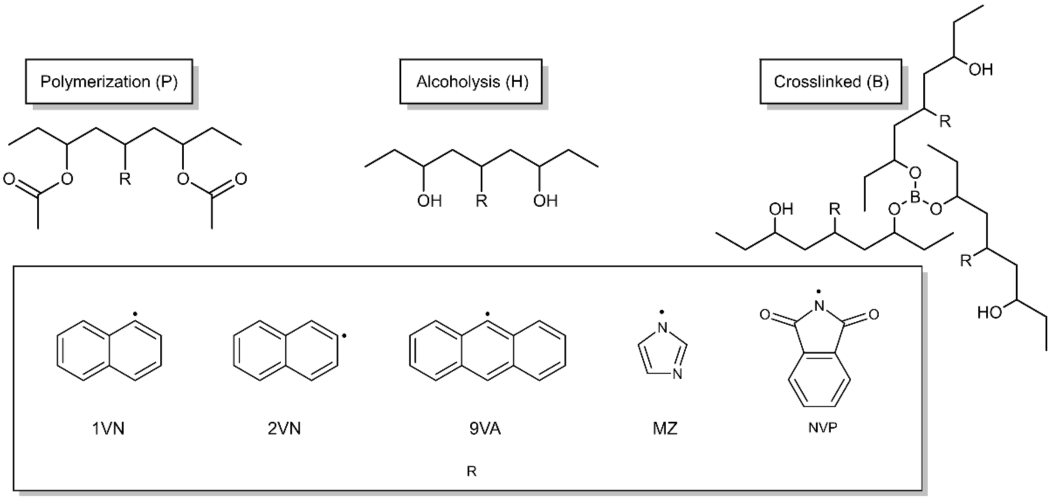

Supplementary Figure 2. Simplified models of the polymeric systems in this work.

Characterization

Thermogravimetric analysis (TGA) was carried out on TA Q50 from 30 to 800 ºC with a ramping rate of 10 ºC min^{-1} in a nitrogen atmosphere. TA Q20 was applied to differential scanning calorimetry (DSC) from -20 to 180 ºC with a ramping rate of 10 ºC min^{-1} in a nitrogen atmosphere. FTIR spectra were carried out using a PerkinElmer Fourier transform infrared spectrometer. Powder X-ray diffraction (XRD) measurements were recorded on a PANalytical Empyrean Series 2 using Cu Kα radiation with 2θ range of 5-60°, 40 KeV, and 40 mA having a scanning rate of 0.026° s^{-1} (2θ) at room temperature. The molecular weight was obtained by gel permeation chromatography (GPC) on Wasters-1525 and Wasters-2414 with water as the mobile phase. 1H NMR was performed on AVANCE III HD 400MHz in deuterated dimethyl sulfoxide (DMSO-*d6*). The phosphorescence spectra and lifetime, steady state and time-resolved emission spectra, and temperature-dependent photoluminescence spectra were measured on Edinburgh FLS 1000 fluorescence spectrophotometer equipped with a xenon lamp (450 W), a laser device (280 nm), and a microsecond flash-lamp (100 W). excitation-dependent emission spectra were measured on Edinburgh FLS 980. Quantum efficiency was obtained on Edinburgh FLS 1000 fluorescence spectrophotometer equipped with an integrating sphere. The lifetime (τ) of the luminescence was obtained by fitting the decay curve with a multi-exponential decay function of equition (6):

$$I(t)=\sum_i A_i e^{-\frac{t}{\tau_i}} \qquad (6)$$

where A_i and τ_i represent the amplitudes and lifetimes of the individual components for multiexponential decay profiles, respectively. The phosphorescence lifetime was analyzed using Exponential Tail Fit analysis that contained a single decay measurement; The photoluminescence lifetime was analyzed using Exponential Reconvolution Fit analysis that contained a single decay and the instrumental response function (IRF). The X-ray photoelectron spectroscopy (XPS) measurements were performed using a Thermo ESCALAB 250 spectrometer with monochromatized Al Kα excitation. Thermogravimetric-infrared (TG-IR) spectra were carried out on NETZSCH TG 209 F1Libra and Nicolet 6700 with a ramping rate of 10 ºC min^{-1} in a nitrogen atmosphere from 30 ºC to 700 ºC. The photos and supporting videos were recorded by a Canon EOS 80D camera.

Supplementary Figures

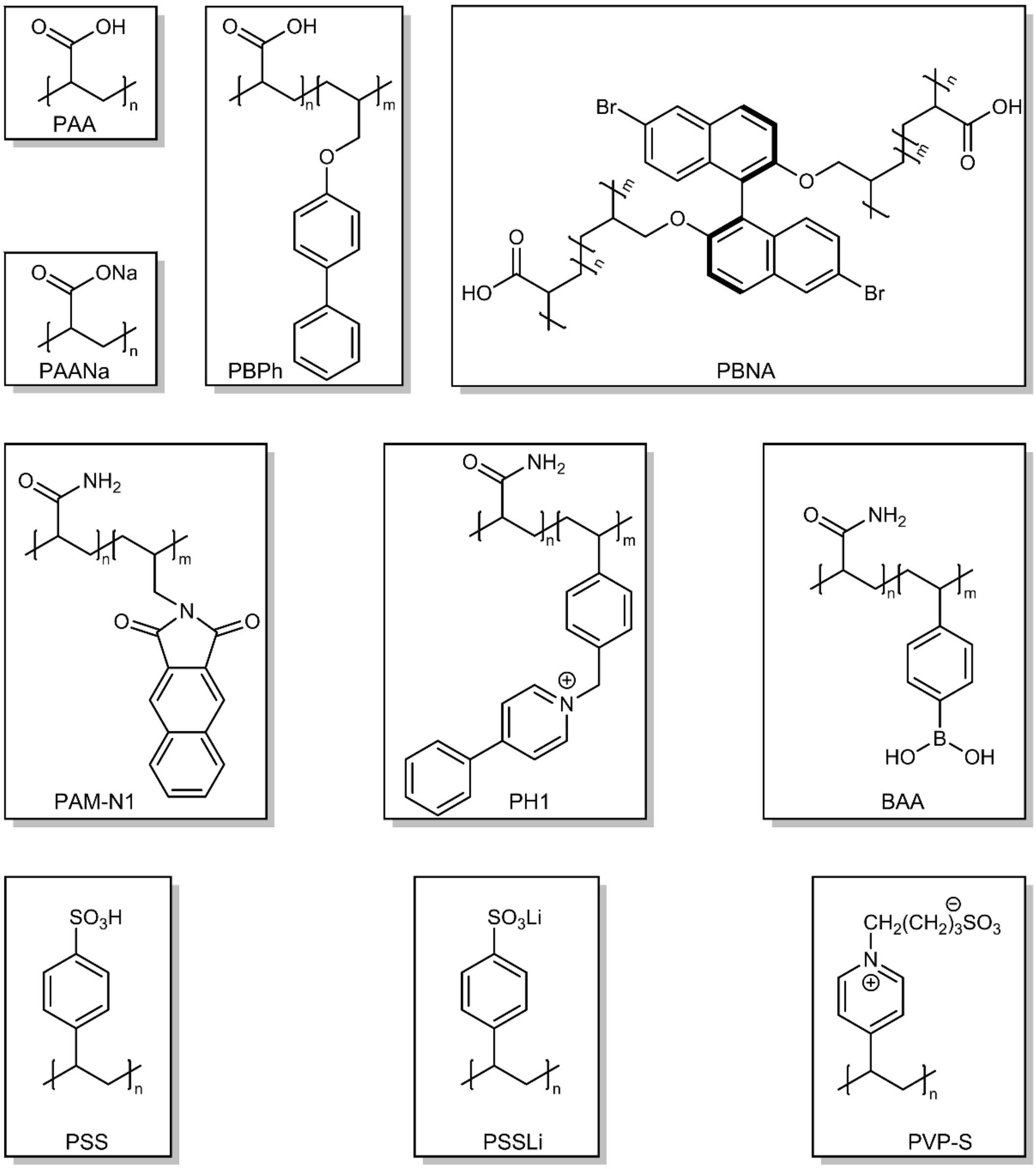

Supplementary Figure 3. Structures of the copolymers with functionalized phosphor groups to develop a range of ambient RTP materials. PAA: polyacrylic acid; PAM: polyacrylamide; PSS: polystyrene sulfonic acid; PVP: polyvinyl pyridine.

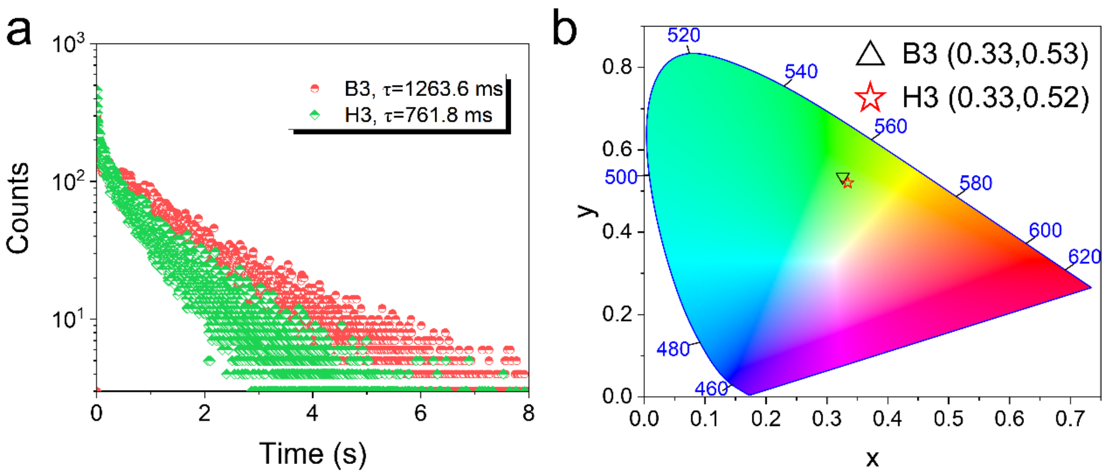

Supplementary Figure 4. Decay spectra and fitted lifetime as well as CIE coordinates. a Decay spectra and fitted lifetime of B3 and H3 at room temperature with λ_{ex} = 300 nm, λ_{em} = 520 nm. **b** CIE coordinates of the phosphorescence from B3 and H3 exhibiting a yellow-green afterglow.

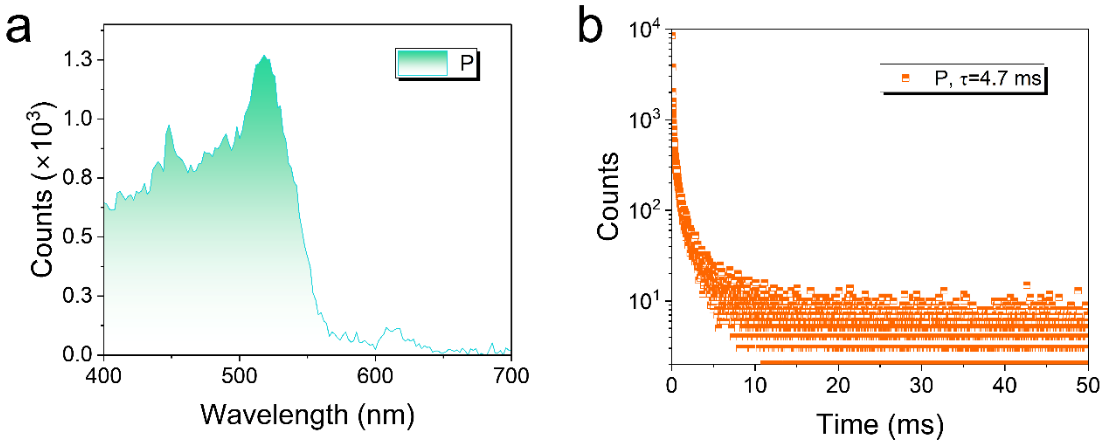

Supplementary Figure 5. Isolated phosphoresce and decay spectra. a,b Isolated phosphoresce (**a**) and decay (**b**) spectra of P3 at room temperature with λ_{ex} = 300 nm, λ_{em} = 518 nm. Phosphorescence was delayed 5 ms.

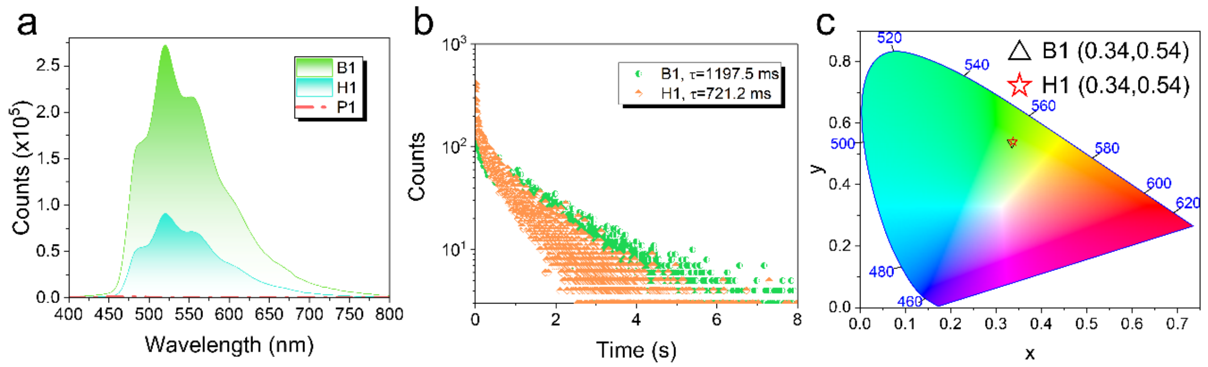

Supplementary Figure 6. RTP emission as well as decay spectra and fitted lifetime. a RTP emission spectra were collected under the same condition with λ_{ex} = 300 nm delayed 5 ms at room temperature. **b** Decay spectra and fitted lifetime of B1 and H1 at room temperature with λ_{ex} = 300 nm, λ_{em} = 520 nm. **c** CIE coordinates of the phosphorescence from B1 and H1 exhibiting a yellow-green afterglow.

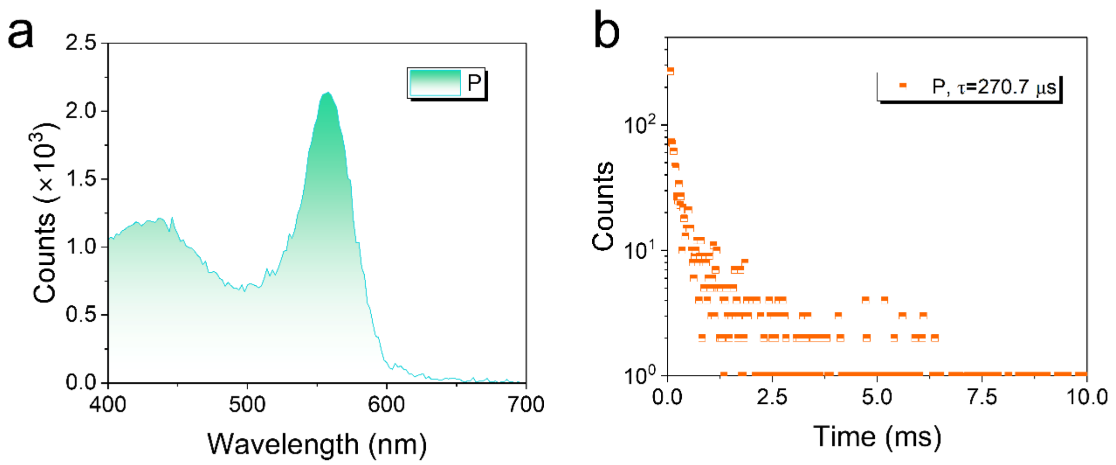

Supplementary Figure 7. Isolated phosphoresce and decay spectra. a,b Isolated phosphoresce (**a**) and decay (**b**) spectra of P1 at room temperature with λ_{ex} = 300 nm, λ_{em} = 558 nm. Phosphorescence was delayed 5 ms.

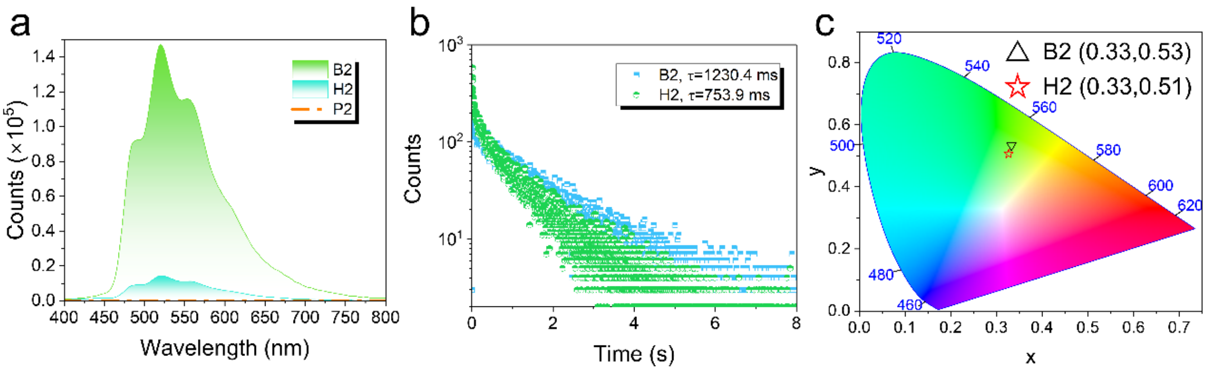

Supplementary Figure 8. RTP emission as well as decay spectra and fitted lifetime. a RTP emission spectra were collected under the same condition with λ_{ex} = 300 nm delayed 5 ms at room temperature. **b** Decay spectra and fitted lifetime of B2 and H2 at room temperature with λ_{ex} = 300 nm, λ_{em} = 520 nm. **c** CIE coordinates of the phosphorescence from B2 and H2 exhibiting a yellow-green afterglow.

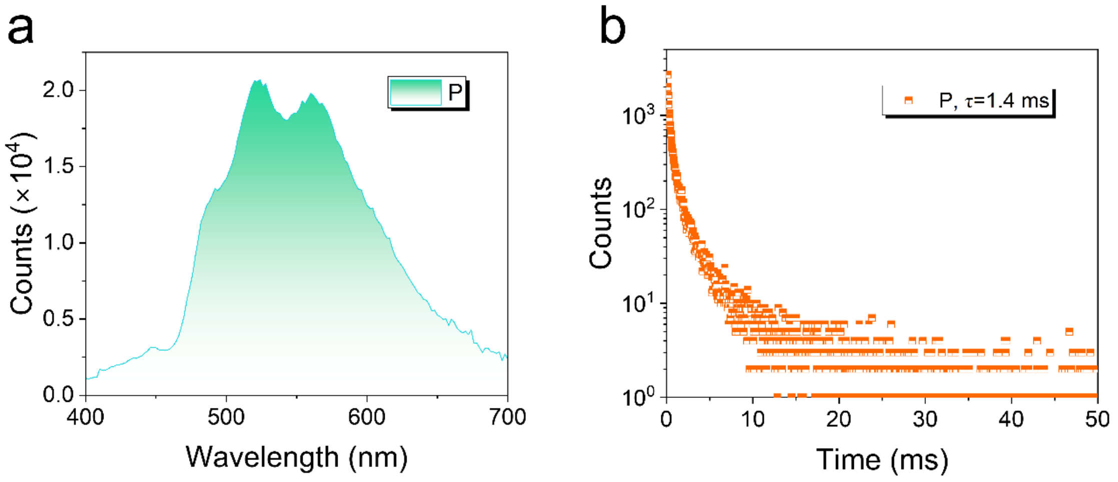

Supplementary Figure 9. Isolated phosphoresce and decay spectra. a,b Isolated phosphoresce (**a**) and decay (**b**) spectra of P2 at room temperature with λ_{ex} = 300 nm, λ_{em} = 522 nm. Phosphorescence was delayed 5 ms.

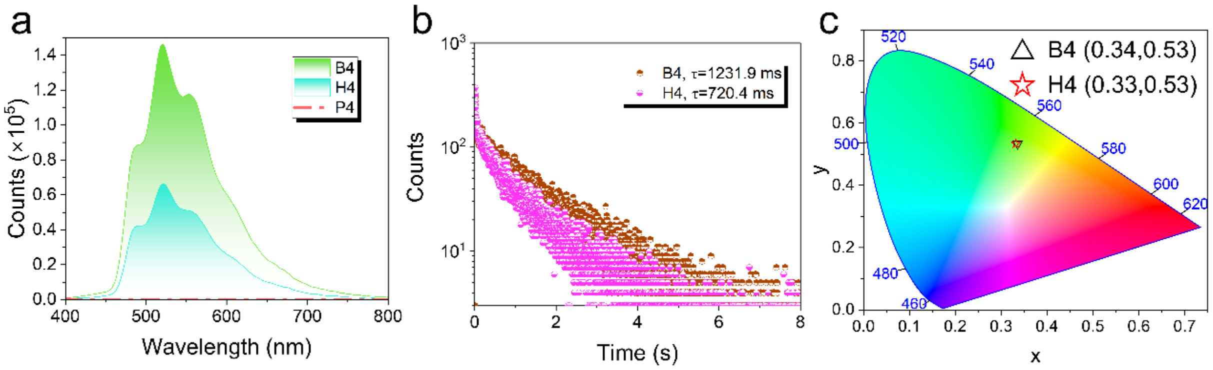

Supplementary Figure 10. RTP emission as well as decay spectra and fitted lifetime. a RTP emission spectra were collected under the same condition with λ_{ex} = 300 nm delayed 5 ms at room temperature. **b** Decay spectra and fitted lifetime of B2 and H2 at room temperature with λ_{ex} = 300 nm, λ_{em} = 520 nm. **c** CIE coordinates of the phosphorescence from B4 and H4 exhibiting a yellow-green afterglow.

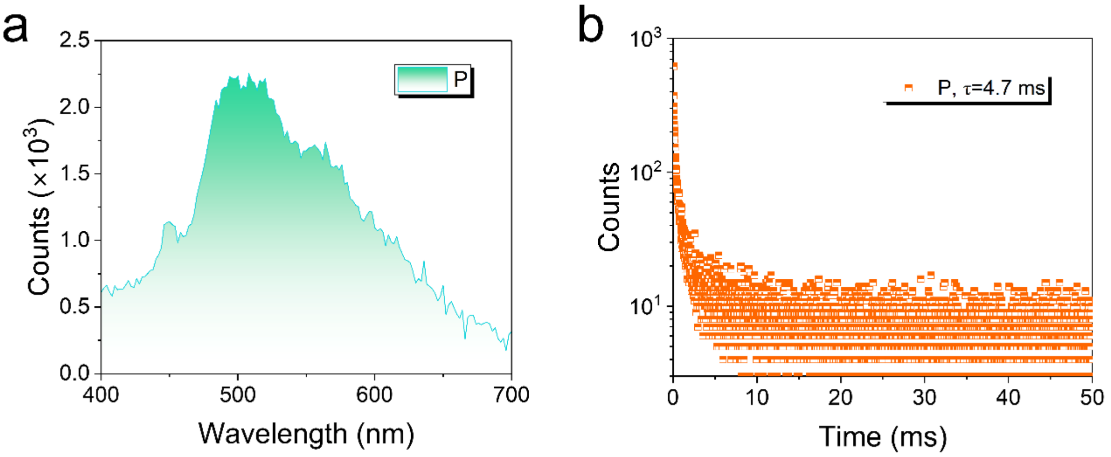

Supplementary Figure 11. Isolated phosphoresce and decay spectra. a,b Isolated phosphoresce (**a**) and decay (**b**) spectra of P4 at room temperature with λ_{ex} = 300 nm, λ_{em} = 508 nm. Phosphorescence was delayed 5 ms.

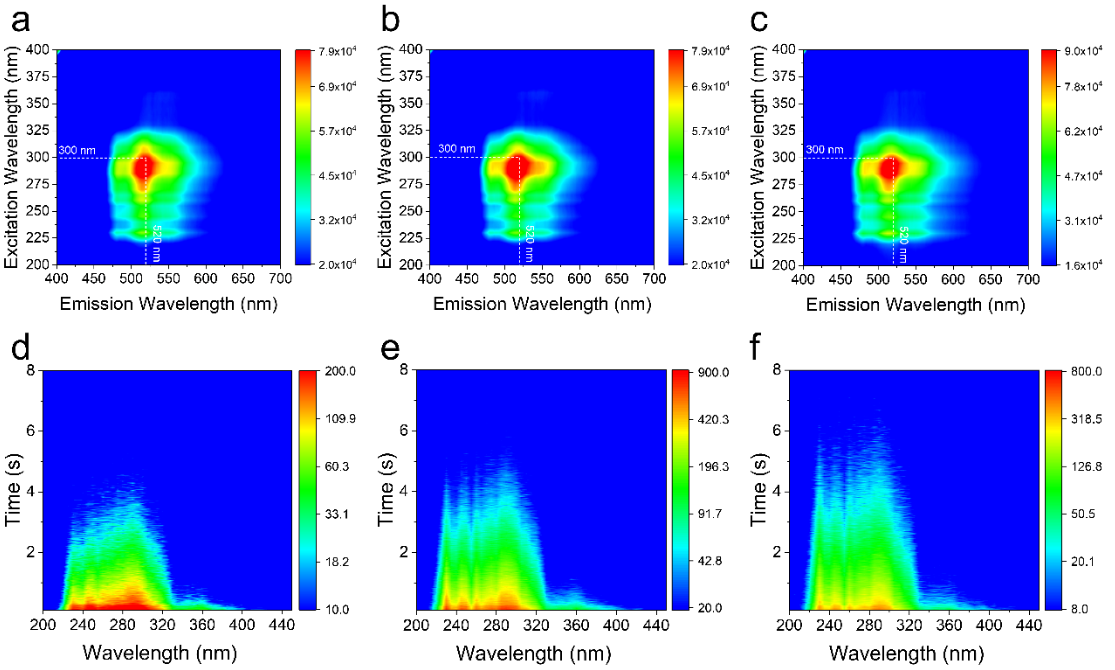

Supplementary Figure 12. Two-dimension emission and excitation spectra. a-c Two-dimension excitation-dependent emission spectra of B1 (**a**), B2 (**b**), and B4 (**c**). **d-f** Two-dimension time resolution excitation spectra of B1 (**d**), B2 (**e**), and B4 (**f**) at λ_{em} = 520 nm.

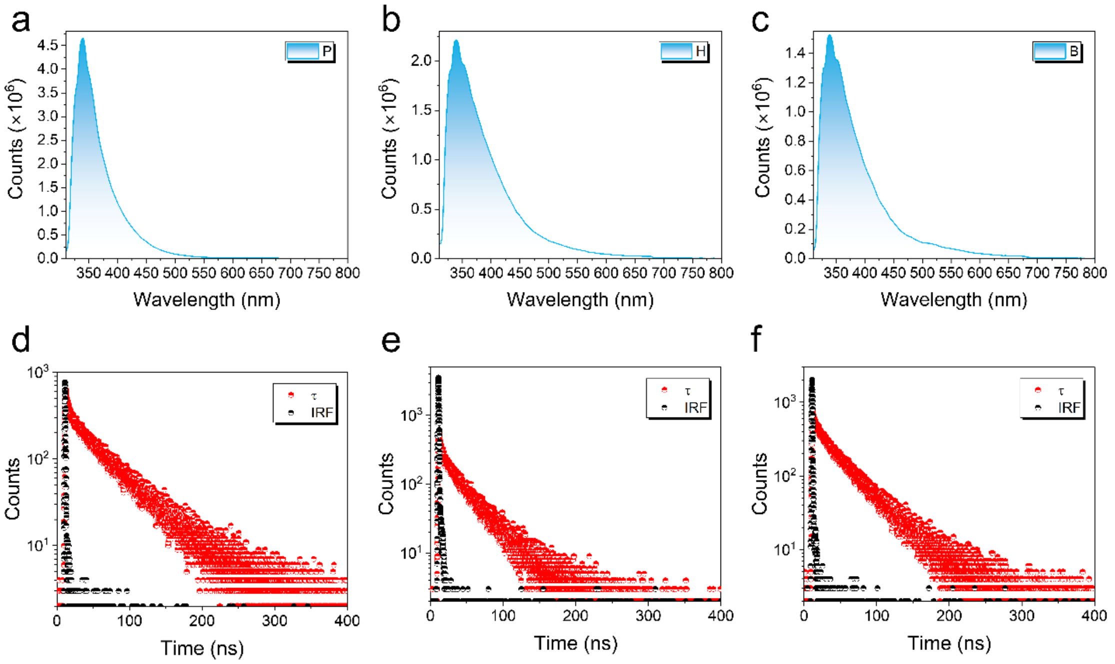

Supplementary Figure 13. Photoluminescence spectra and decay spectra. a-c Photoluminescence spectra at λ_{ex} = 300 nm for P1 (**a**), H1 (**b**), and B1 (**c**). **d-f** Photoluminescence decay spectra with IRF at λ_{em} = 340 nm, 340 nm, and 338 nm for P1 (**d**), H1 (**e**), and B1 (**f**).

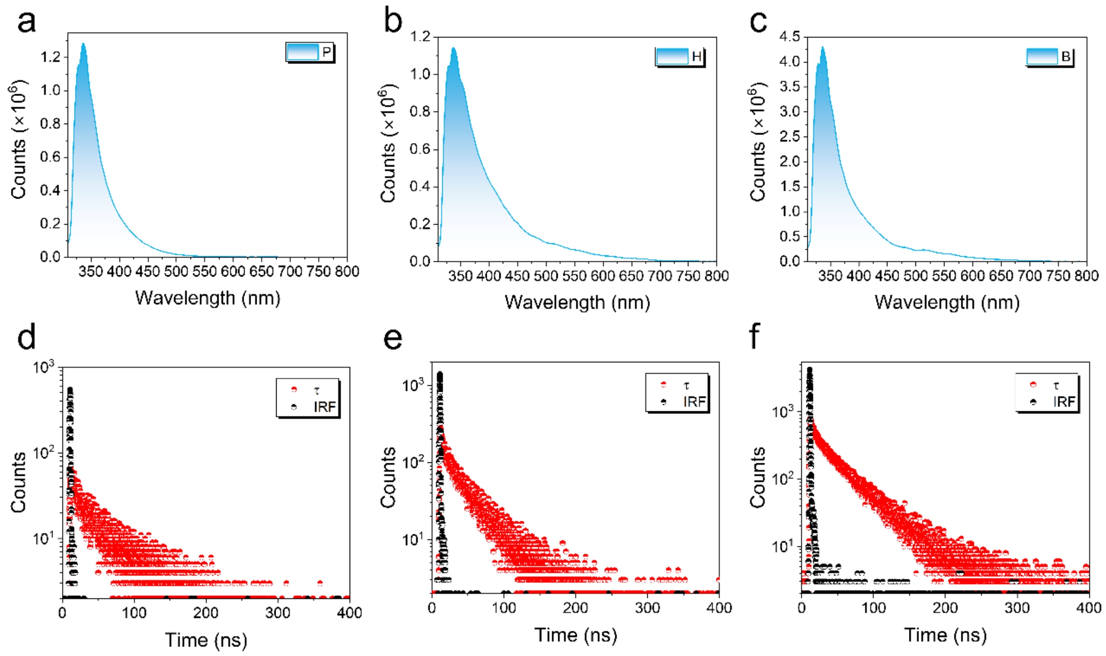

Supplementary Figure 14. Photoluminescence spectra and decay spectra. a-c Photoluminescence spectra at λ_{ex} = 300 nm for P2 (**a**), H2 (**b**), and B2 (**c**). **d-f** Photoluminescence decay spectra with IRF at λ_{em} = 336 nm, 338 nm, and 336 nm for P2 (**d**), H2 (**e**), and B2 (**f**).

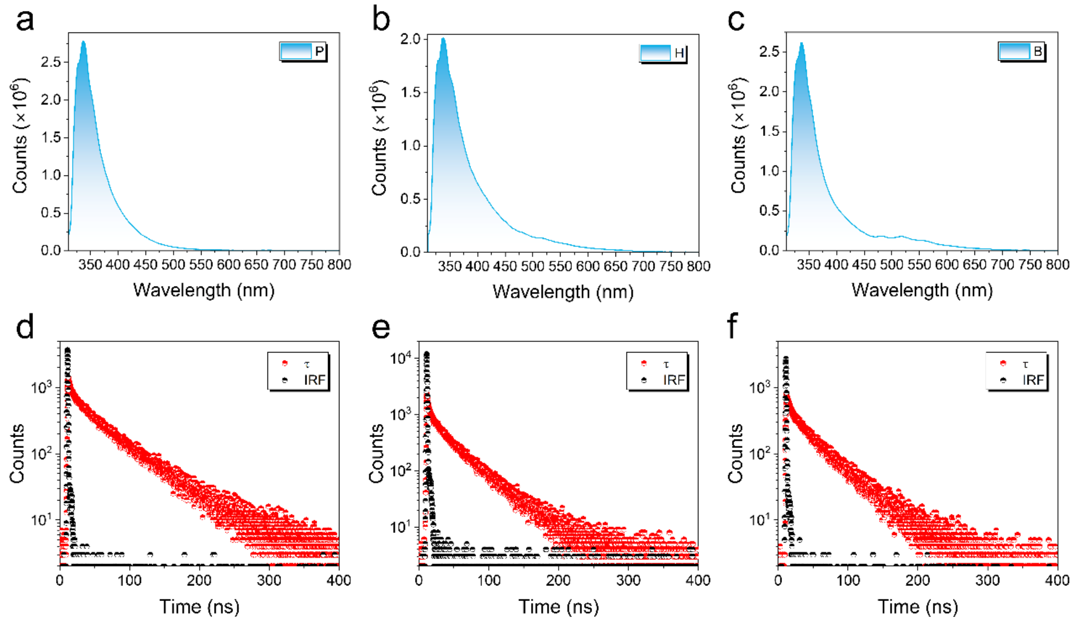

Supplementary Figure 15. Photoluminescence spectra and decay spectra. a-c Photoluminescence spectra at λ_{ex} = 300 nm for P3 (**a**), H3 (**b**), and B3 (**c**). **d-f** Photoluminescence decay spectra with IRF at λ_{em} = 338 nm, 338 nm, and 336 nm for P3 (**d**), H3 (**e**), and B3 (**f**).

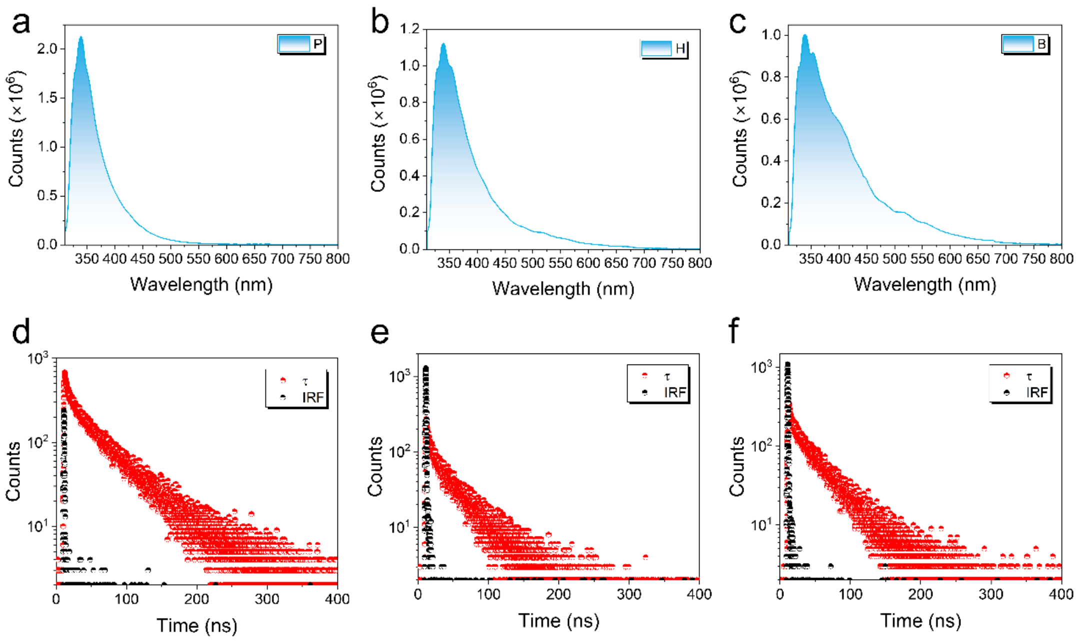

Supplementary Figure 16. Photoluminescence spectra and decay spectra. a-c Photoluminescence spectra at λ_{ex} = 300 nm for P4 (**a**), H4 (**b**), and B4 (**c**). **d-f** Photoluminescence decay spectra with IRF at λ_{em} = 340 nm, 340 nm, and 340 nm for P4 (**d**), H4 (**e**), and B4 (**f**).

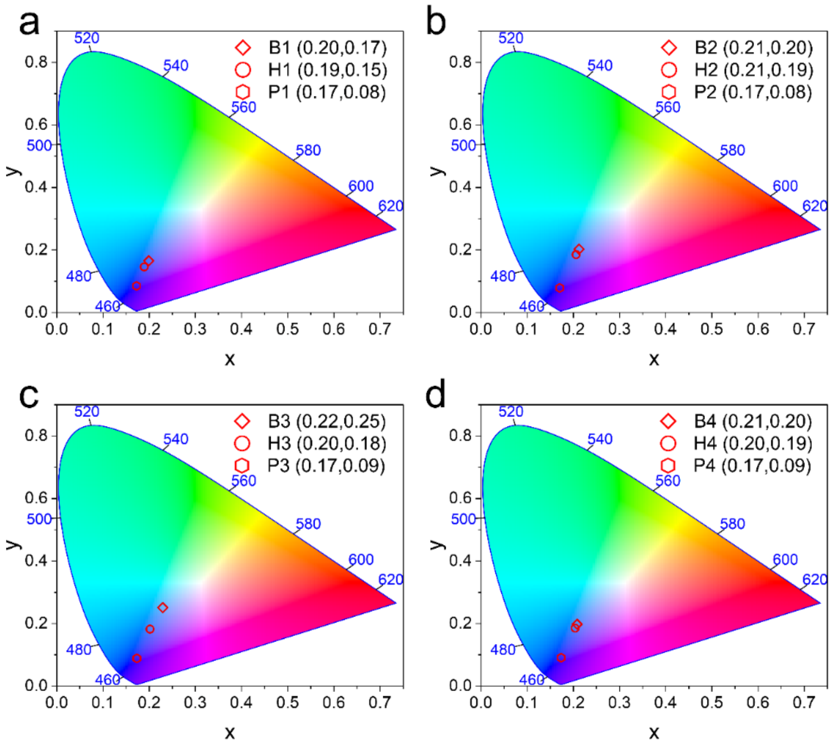

Supplementary Figure 17. CIE coordinates. a-d CIE coordinates from the photoluminescence spectra exhibiting a blue color.

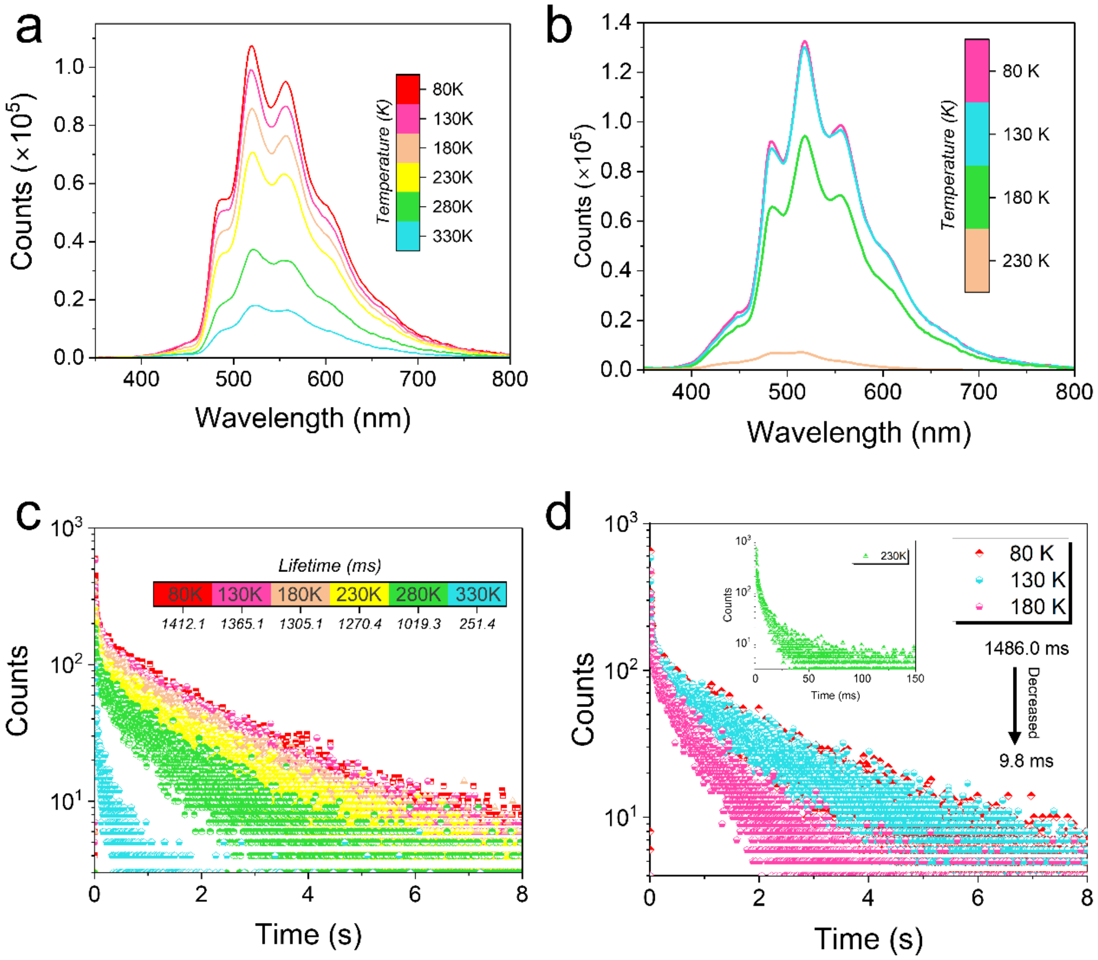

Supplementary Figure 18. Temperature-dependent phosphorescence as well as decay spectra and fitted lifetime. a,b Temperature-dependent phosphorescence of H3 (**a**) and P3 (**b**) at 520 nm excited by 300 nm µF lamp (delayed 5 ms). **c,d** Decay spectra and fitted lifetime from H3 (**c**) and P3 (**d**).

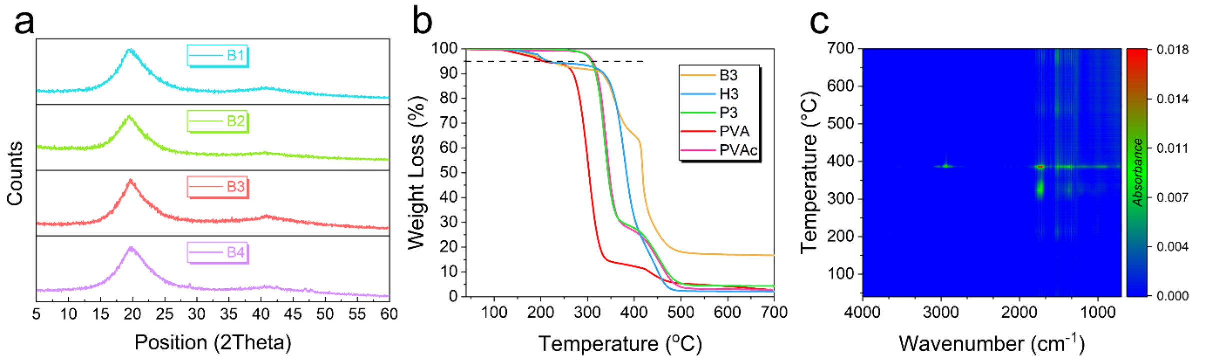

Supplementary Figure 19. XRD, TGA and TG-IR. **a** Powder X-ray diffraction (XRD) of B1–B4 at room temperature. **b** Thermogravimetric analysis (TGA) of P3, H3, and B3 compared with PVA and PVAc (dashed line indicates 95% weight loss): the decomposition behavior of P3 is consistent with that of PVAc. The decomposition behavior of B3 and H3 is similar to the PVA. **c** TG-IR analysis of B3 from 30 °C to 700 °C: the violently decomposition of B3 begins at 300 °C.

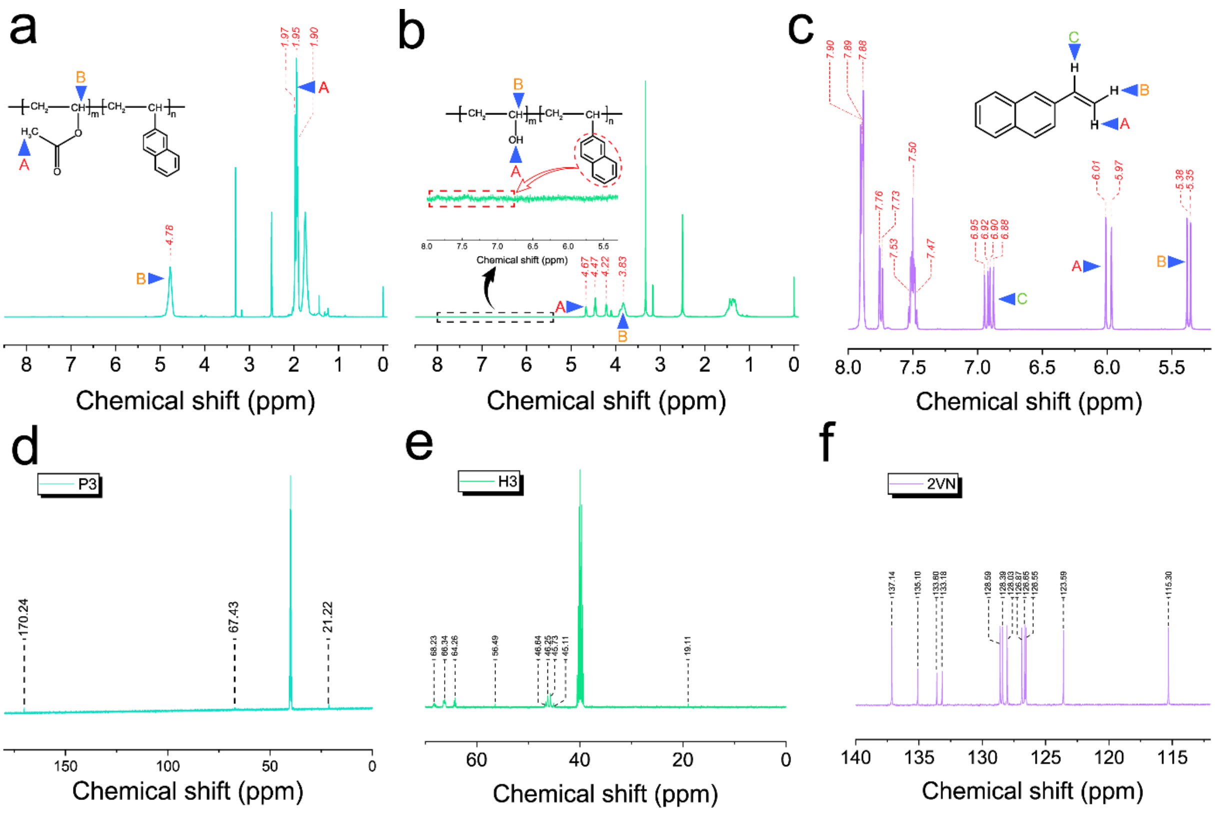

Supplementary Figure 20. ^{1}H and ^{13}C NMR spectra. a-c ^{1}H NMR spectra of P3 (**a**), H3 (**b**), and 2VN (**c**) obtained from DMSO-d_6. **d-f** ^{13}C NMR spectra of P3 (**d**), H3 (**e**), and 2VN (**f**) obtained from DMSO-d_6.

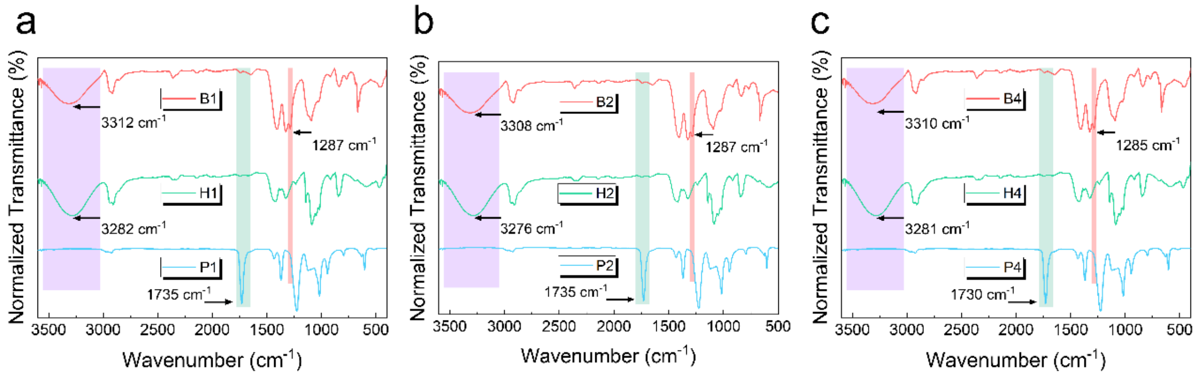

Supplementary Figure 21. IR spectra. a-c Comparison of IR spectra from polymerization (P), alcoholysis (H), and after cross-linking (B).

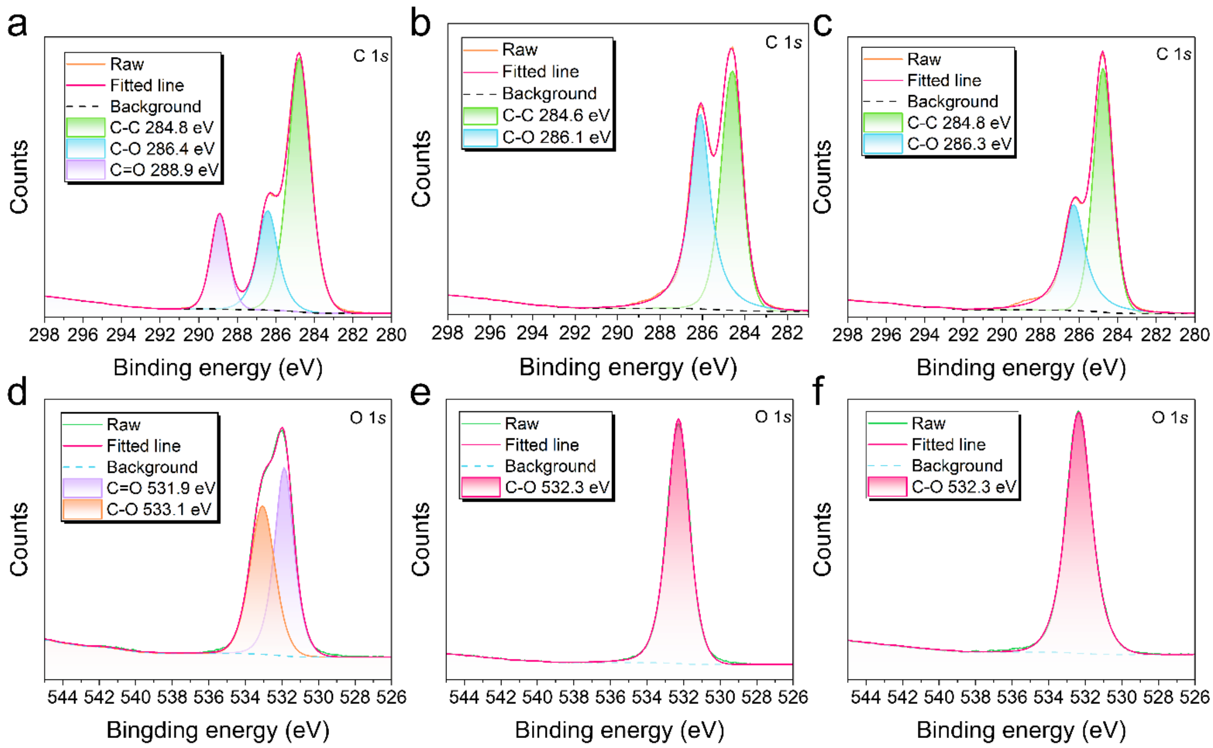

Supplementary Figure 22. XPS spectra. a-f Comparison of XPS high-resolution C 1s and O 1s from P3 (**a,d**), H3 (**b,e**), and B3 (**c,f**).

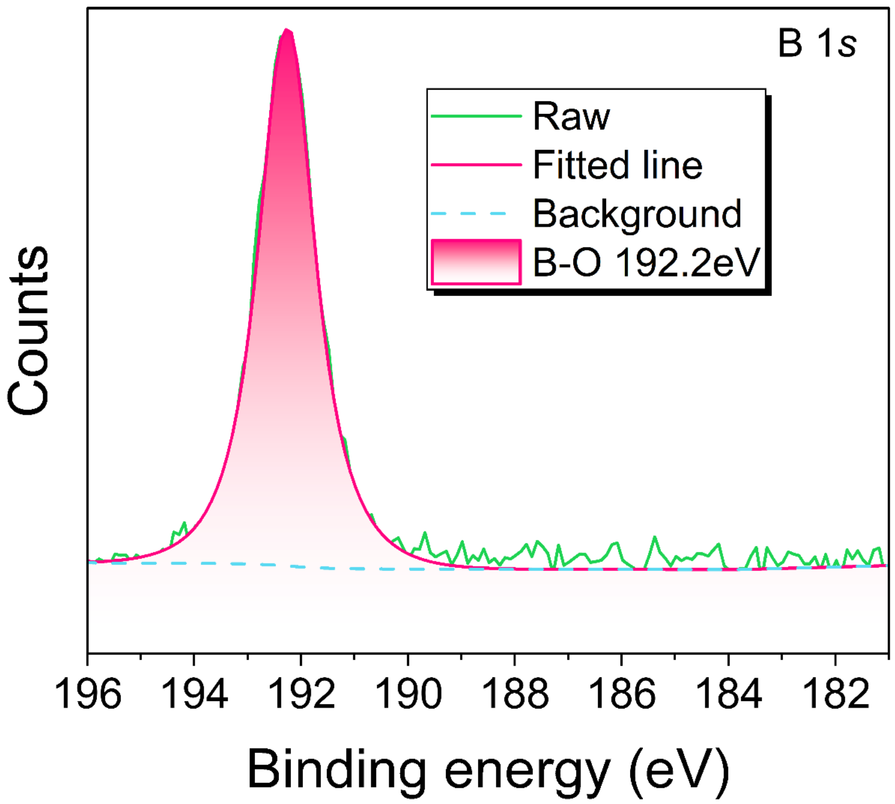

Supplementary Figure 23. XPS high-resolution B1s spectra of B3.

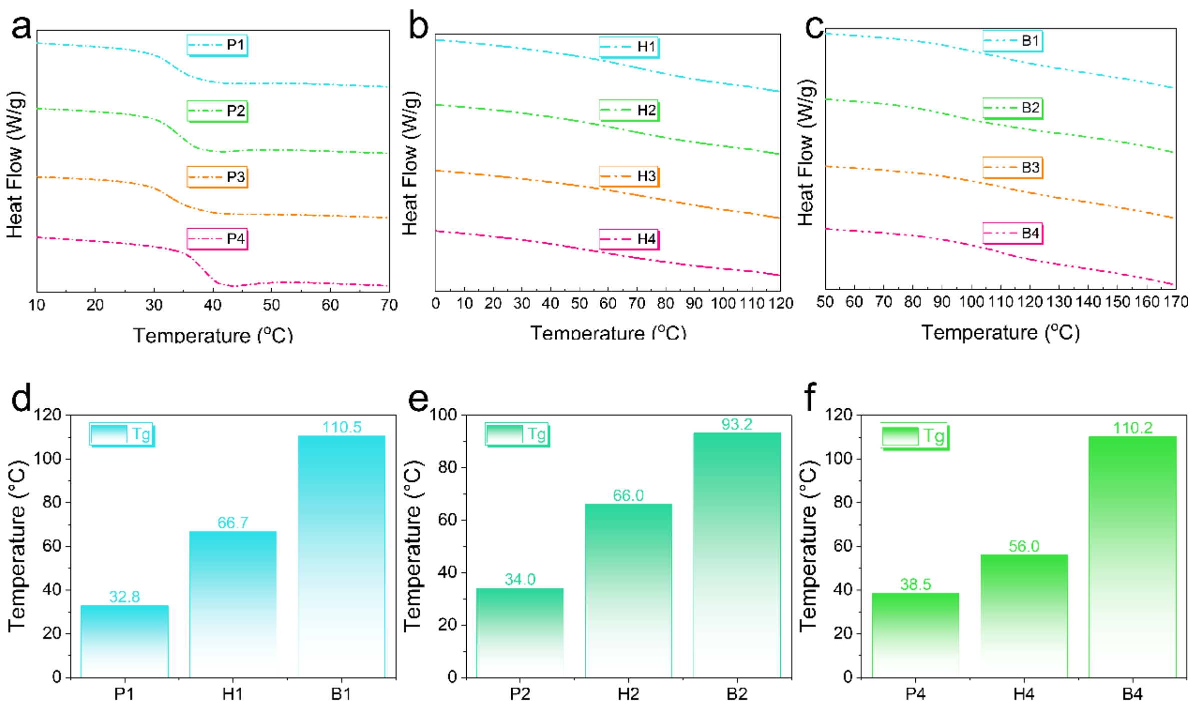

Supplementary Figure 24. DSC results. a-c DSC curves of polymerization (**a**), alcoholysis (**b**), and after cross-linking (**c**). **d-f** Comparison of Tg from P1 to B1 (**d**), P2 to B2 (**e**), and P4 to B4 (**f**).

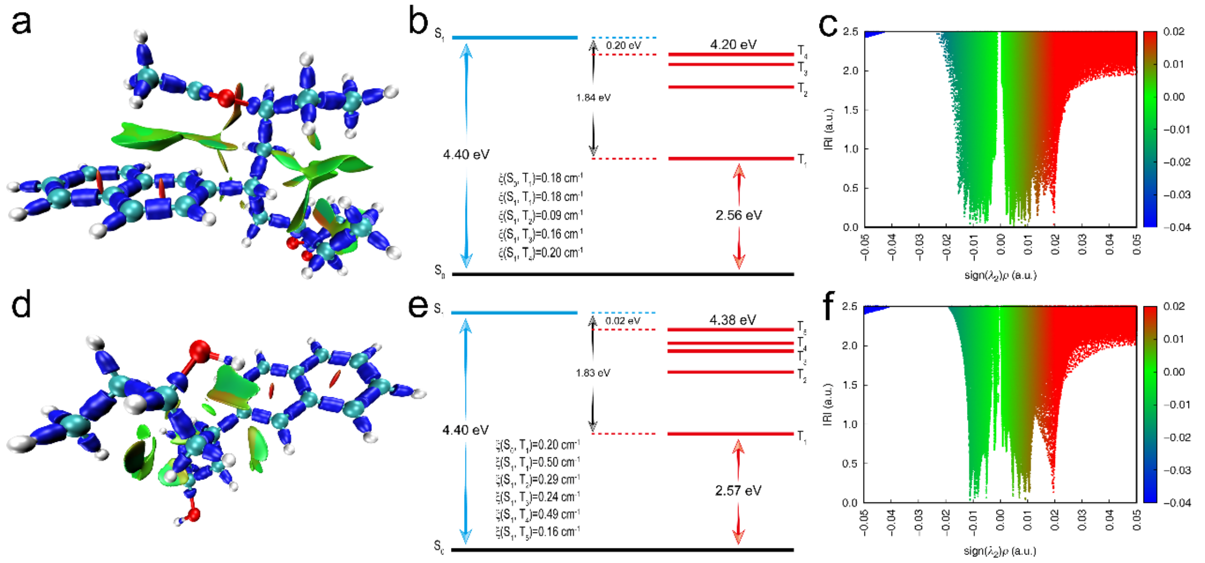

Supplementary Figure 25. Calculation studies. a-f Visualization of interactions in polymerization (**a**), and alcoholysis (**d**) structures; Theoretical calculation of the optimized simplified model: vertical excitation energy of polymerization (**b**), and alcoholysis (**e**) with boosted RTP performance: vertical excitation energies and spin-orbit coupling constant (SOC); Distribution of different interactions via sign $(\lambda_2)\rho$ on IRI isosurfaces in polymerization (**c**), and alcoholysis (**f**).

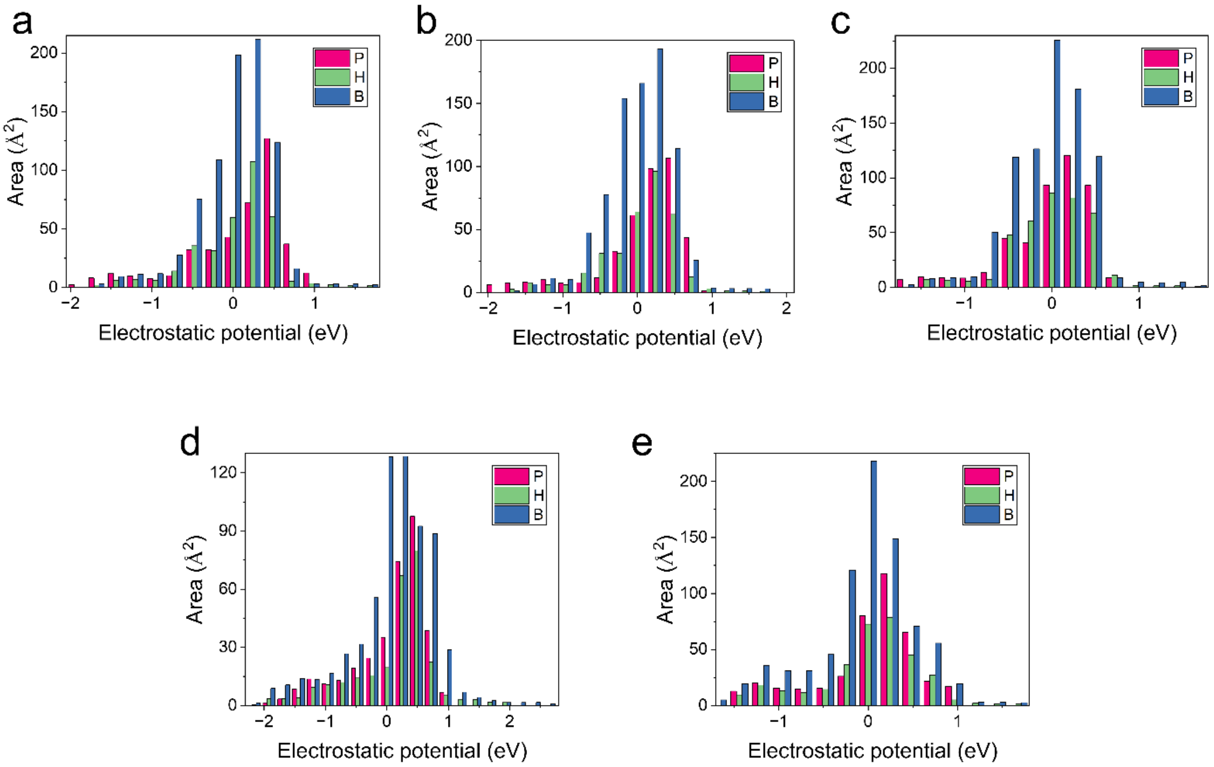

Supplementary Figure 26. Area in ESP. a-d Area in the electrostatic potential (ESP) of polymerization (P), alcoholysis (H), and cross-linked (B) structures from 2VN (**a**), 1VN (**b**), 9VA (**c**), MZ (**d**), and NVP (**e**).

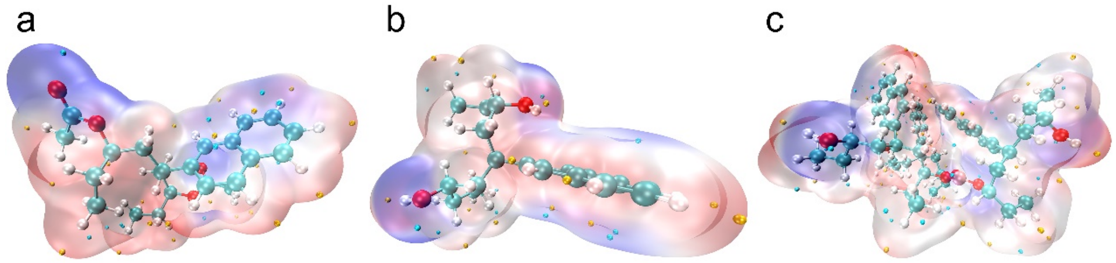

Supplementary Figure 27. Distribution of ESP. a-c Distribution of ESP in polymerization (**a**), alcoholysis (**b**), and cross-linked (**c**) structures. Negative potential (blue), positive potential (red), and neutral potential (white). The balls are the extreme point of regional potential (blue: minimum value; yellow: maximum value).

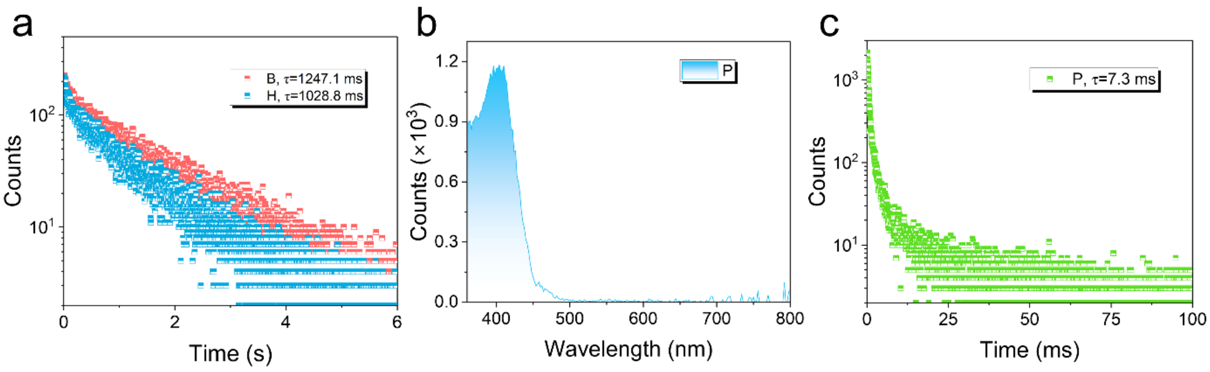

Supplementary Figure 28. Decay spectra, isolated phosphoresce and fitted lifetime. a Decay spectra and fitted lifetime of crosslinked (B) and alcoholysis (H) at room temperature from 1VN. **b,c** Isolated phosphoresce (**b**) and decay (**c**) spectra of polymerization (P) at room temperature with λ_{ex} = 300 nm, λ_{em} = 404 nm. Phosphorescence was delayed 5 ms.

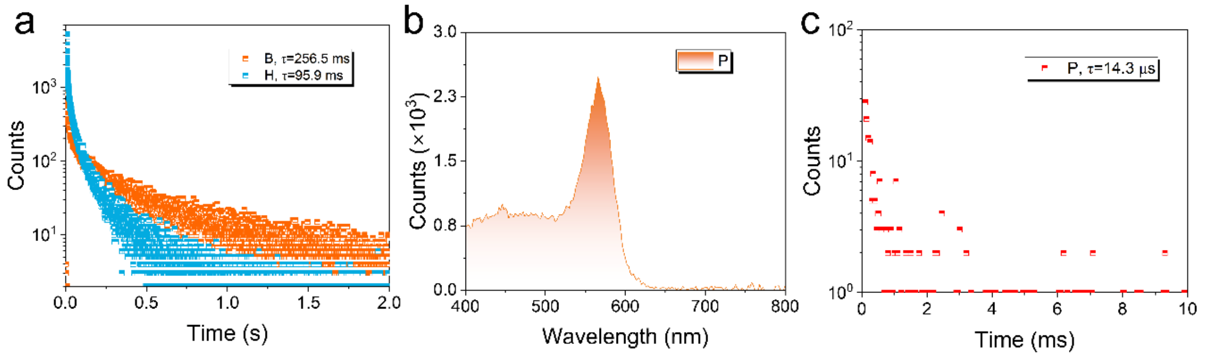

Supplementary Figure 29. Decay spectra, isolated phosphoresce and fitted lifetime. a Decay spectra and fitted lifetime of crosslinked (B) and alcoholysis (H) at room temperature from 9VA. **b,c** Isolated phosphoresce (**b**) and decay (**c**) spectra of polymerization (P) at room temperature with λ_{ex} = 300 nm, λ_{em} = 566 nm. Phosphorescence was delayed 5 ms.

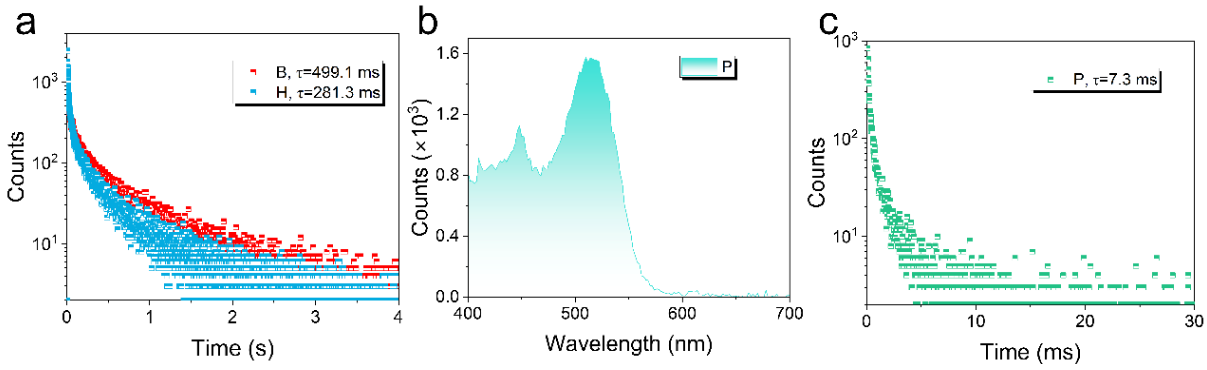

Supplementary Figure 30. Decay spectra, isolated phosphoresce and fitted lifetime. a Decay spectra and fitted lifetime of crosslinked (B) and alcoholysis (H) at room temperature from MZ. **b,c** Isolated phosphoresce (**b**) and decay (**c**) spectra of polymerization (P) at room temperature with λ_{ex} = 300 nm, λ_{em} = 510 nm. Phosphorescence was delayed 5 ms.

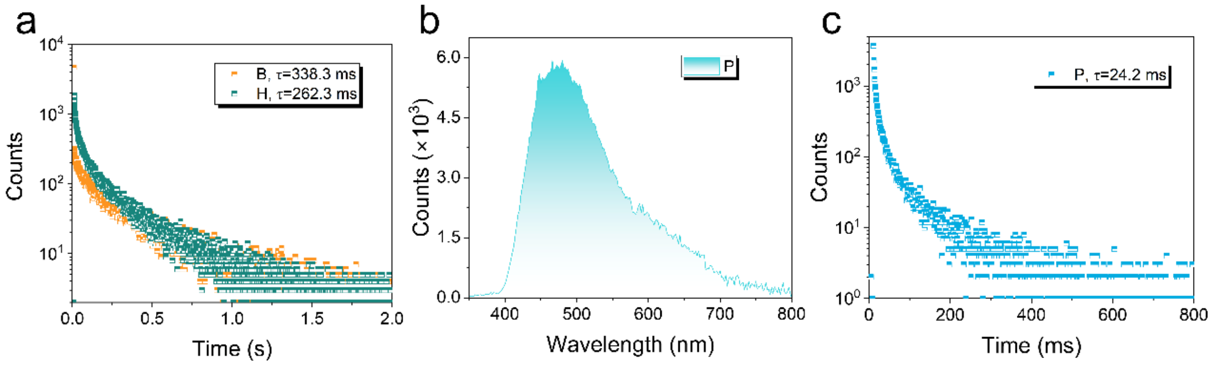

Supplementary Figure 31. Decay spectra, isolated phosphoresce and fitted lifetime. a Decay spectra and fitted lifetime of crosslinked (B) and alcoholysis (H) at room temperature from NVP. **b,c** Isolated phosphoresce (**b**) and decay (**c**) spectra of polymerization (P) at room temperature with λ_{ex} = 300 nm, λ_{em} = 480 nm. Phosphorescence was delayed 5 ms.

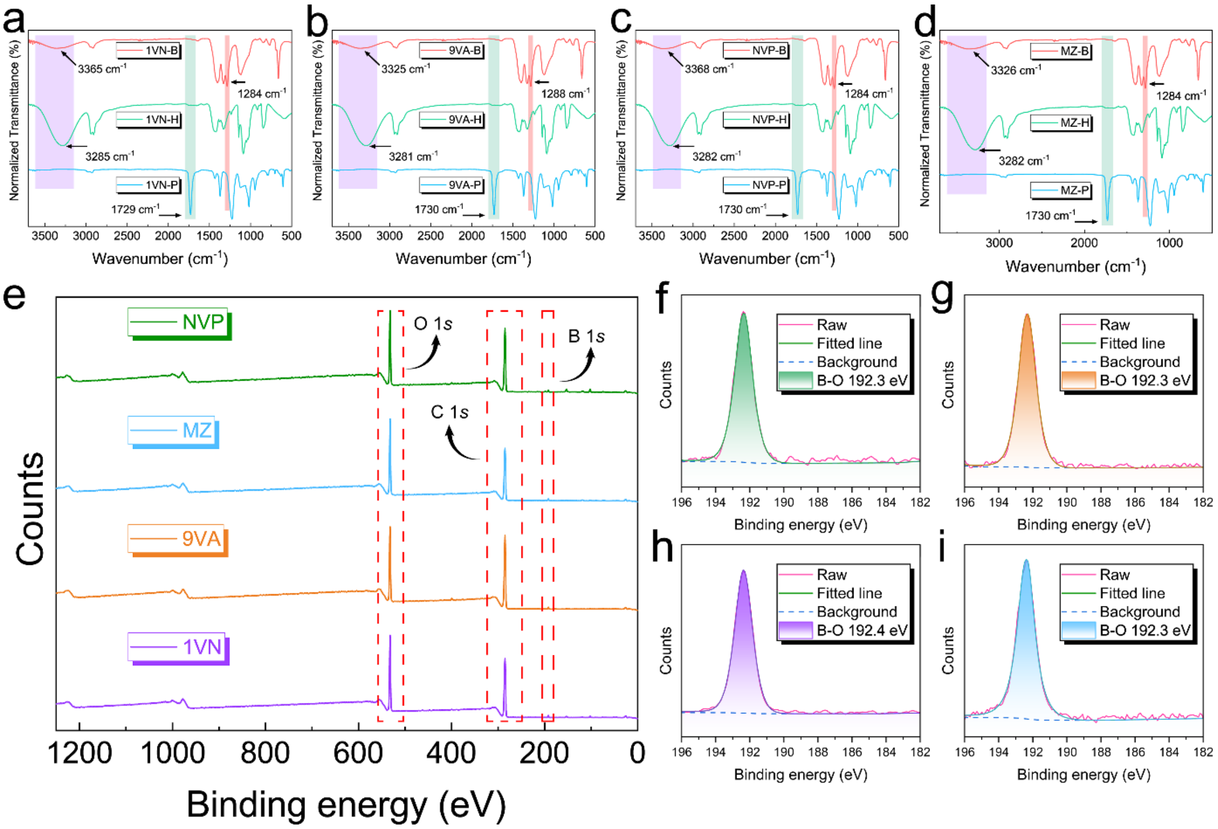

Supplementary Figure 32. IR spectra and XPS spectra. a-d Comparison of IR spectra from polymerization (P), alcoholysis (H), and cross-linked (B) of 1VN (**a**), 9VA (**b**), NVP (**c**), and MZ (**d**). **e** XPS spectra of crosslinked polymer prepared by copolymerization of different phosphorescence units. **f-i** XPS high-resolution B1s spectra of NVP (**f**), 9VA (**g**), 1VN (**h**) and MZ (**i**). The hydrogen bonding vibration at 3281-3285 cm^{-1} is significantly sharper in H than in B, which was undetectable in P3. For instance, the stretching vibration of C=O at 1730 cm^{-1} is stronger in P than in H and B, and the vibration of the hydrogen bond at 3325-3368 cm^{-1} is weaker in B than in H. This indicates that P did not provide as strong of a hydrogen bond and covalent cross-linking as H3 and B3, with the B-O stretching vibration at 1284-1288 cm^{-1} in B.

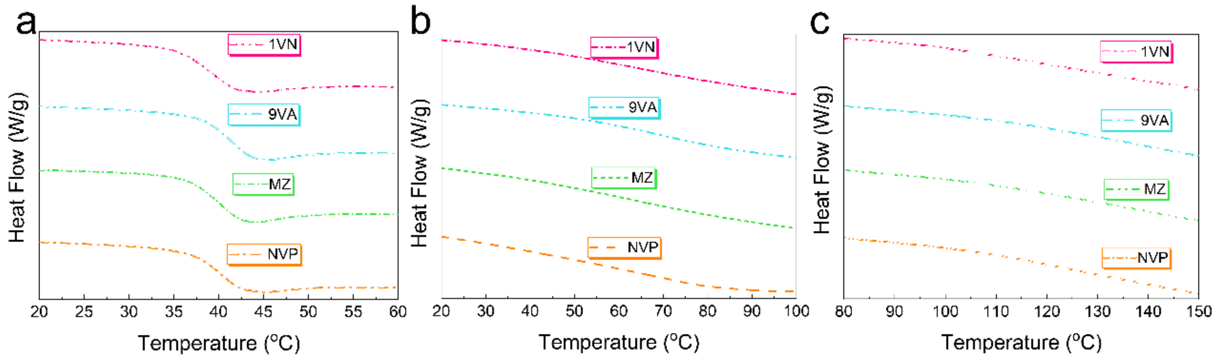

Supplementary Figure 33. DSC curves. a-c DSC curves of polymerization (**a**), alcoholysis (**b**), and after cross-linking (**c**) of different phosphors.

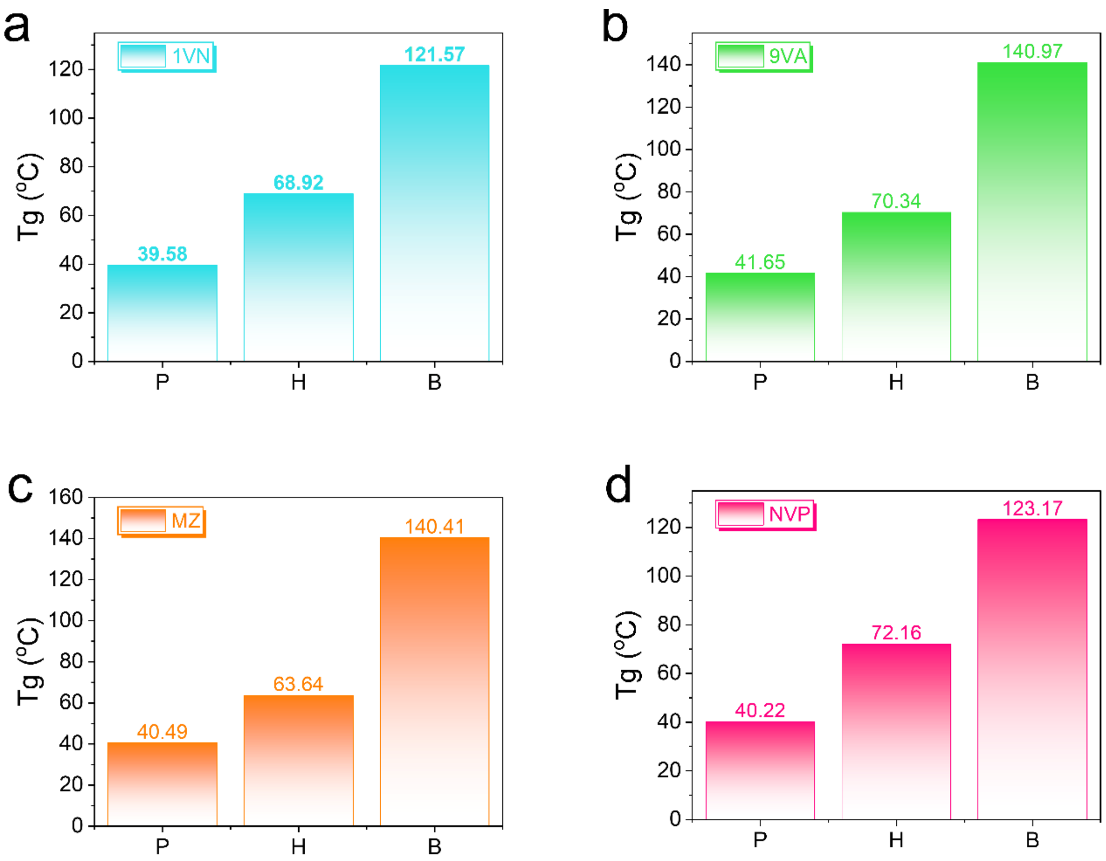

Supplementary Figure 34. Comparison of Tg. a-d Comparison of Tg from P to B of 1VN (**a**), 9VA (**b**), MZ (**c**), and NVP (**d**). In P, where there is no hydrogen bonding and no cross-linking network, Tg is 39.58-41.65 °C. In H, where hydrogen bonding is existent, Tg is 63.64-72.16 °C, and B is reaching 121.57-140.97 °C via cross-linking.

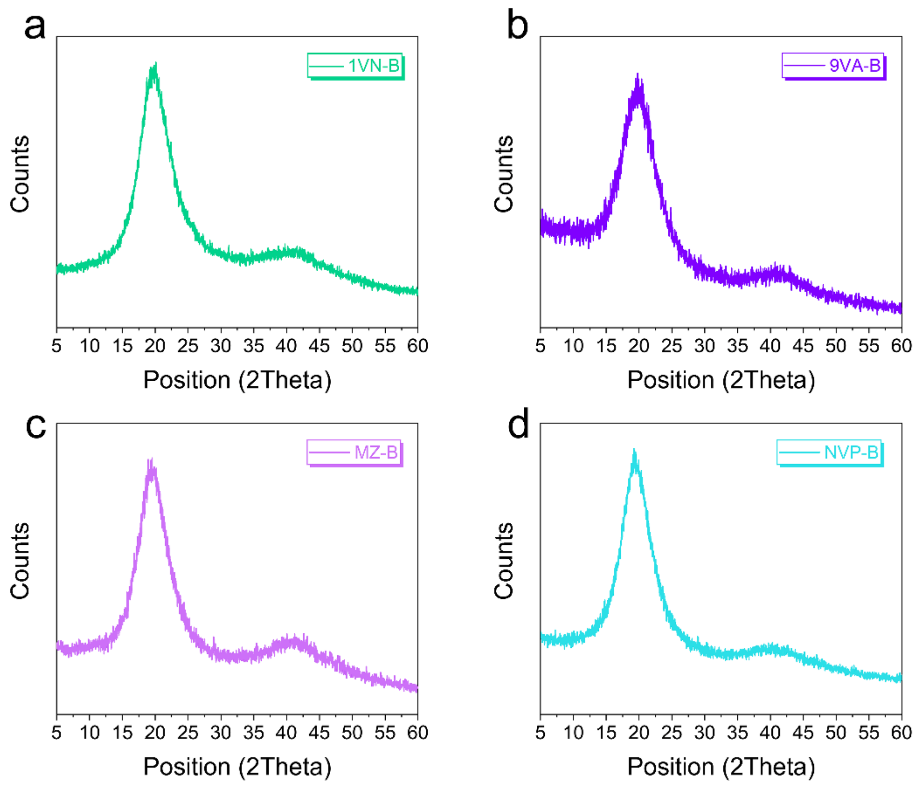

Supplementary Figure 35. XRD patterns. a-d Powder X-ray diffraction (XRD) of cross-linked structure at room temperature from 1VN (**a**), 9VA (**b**), MZ (**c**), and NVP (**d**).

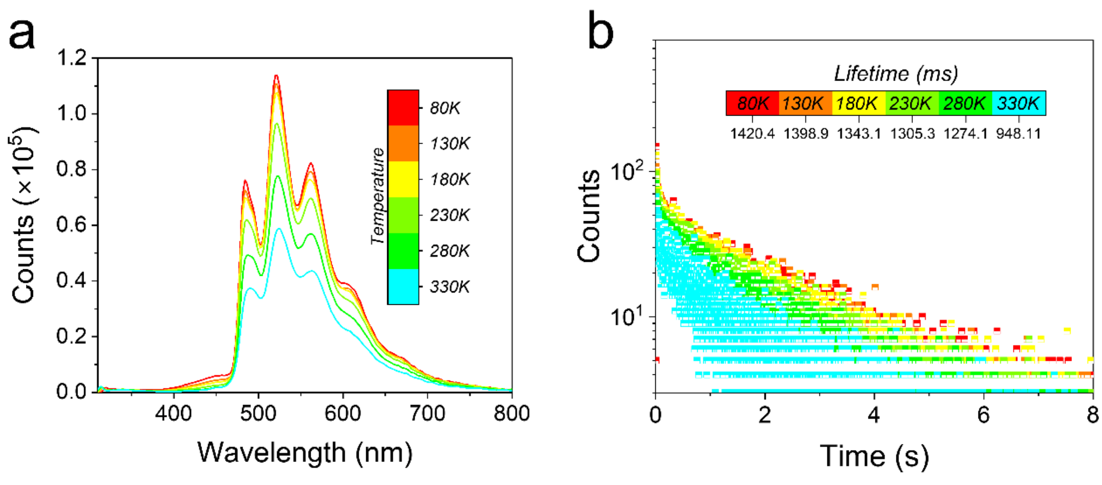

Supplementary Figure 36. Temperature-dependent phosphorescence as well as decay spectra and fitted lifetime. a Temperature-dependent phosphorescence of 1VN-B excited by 300 nm μF lamp (delayed 5 ms). **b** Decay spectra and fitted lifetime from 1VN-B at different temperatures.

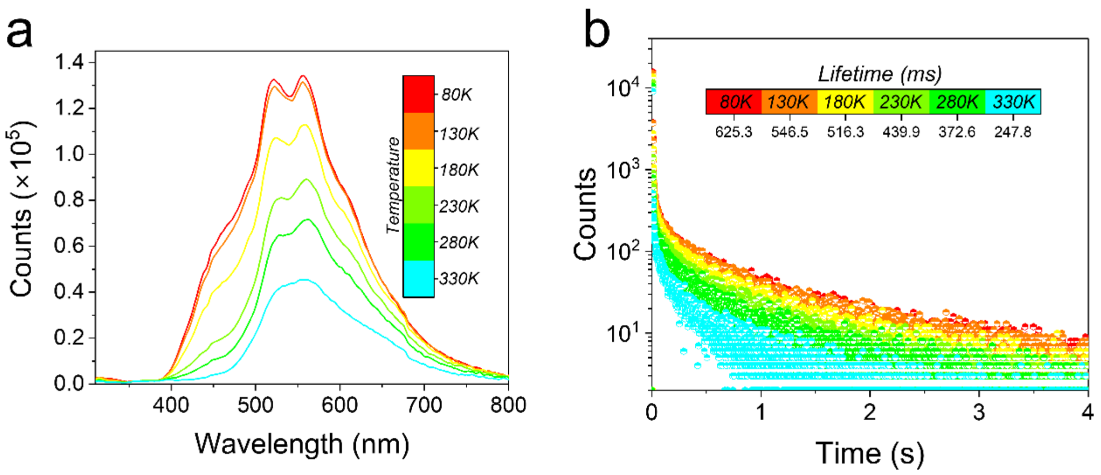

Supplementary Figure 37. Temperature-dependent phosphorescence as well as decay spectra and fitted lifetime. a Temperature-dependent phosphorescence of 9VA-B excited by 300 nm μF lamp (delayed 5 ms). **b** Decay spectra and fitted lifetime from 9VA-B at different temperatures.

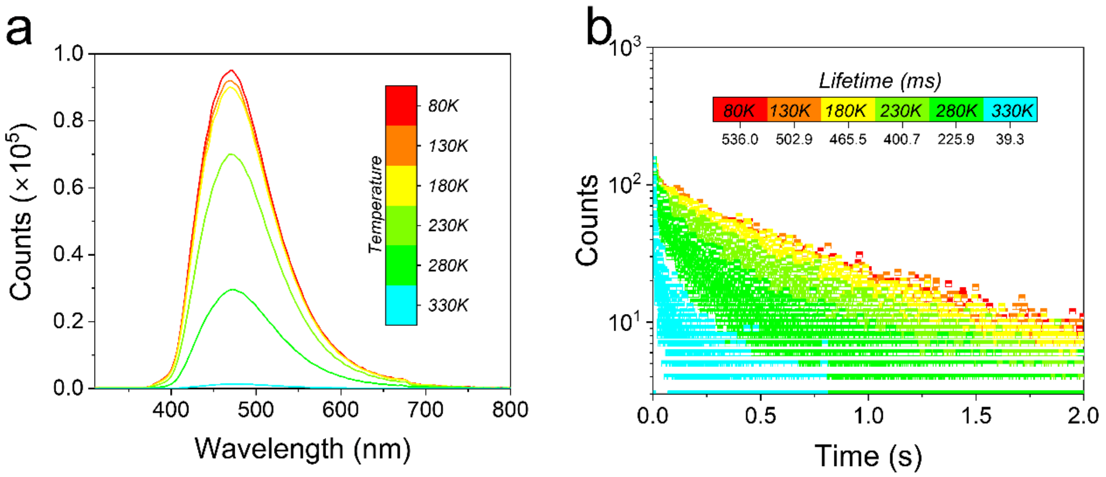

Supplementary Figure 38. Temperature-dependent phosphorescence as well as decay spectra and fitted lifetime. a Temperature-dependent phosphorescence of MZ-B excited by 300 nm μF lamp (delayed 5 ms). **b** Decay spectra and fitted lifetime from MZ-B at different temperatures.

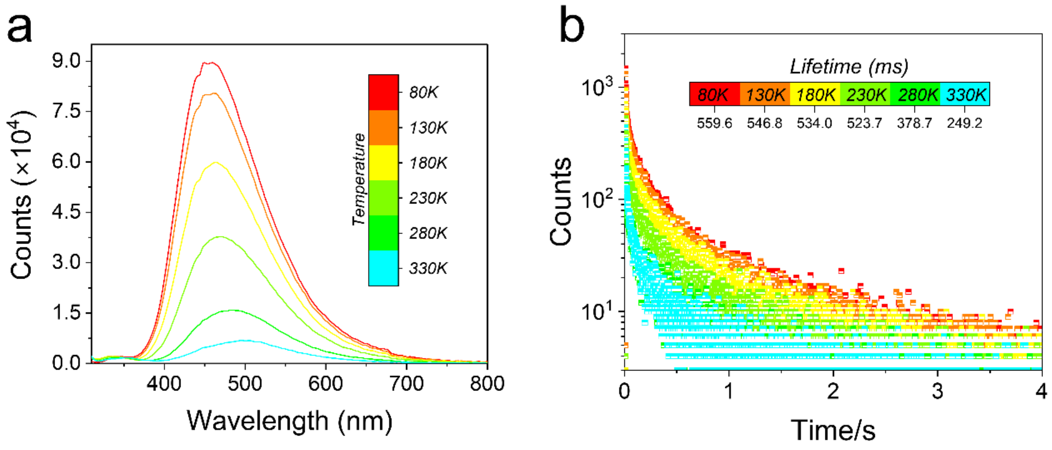

Supplementary Figure 39. Temperature-dependent phosphorescence as well as decay spectra and fitted lifetime. a Temperature-dependent phosphorescence of NVP-B excited by 300 nm μF lamp (delayed 5 ms). **b** Decay spectra and fitted lifetime from NVP-B at different temperatures.

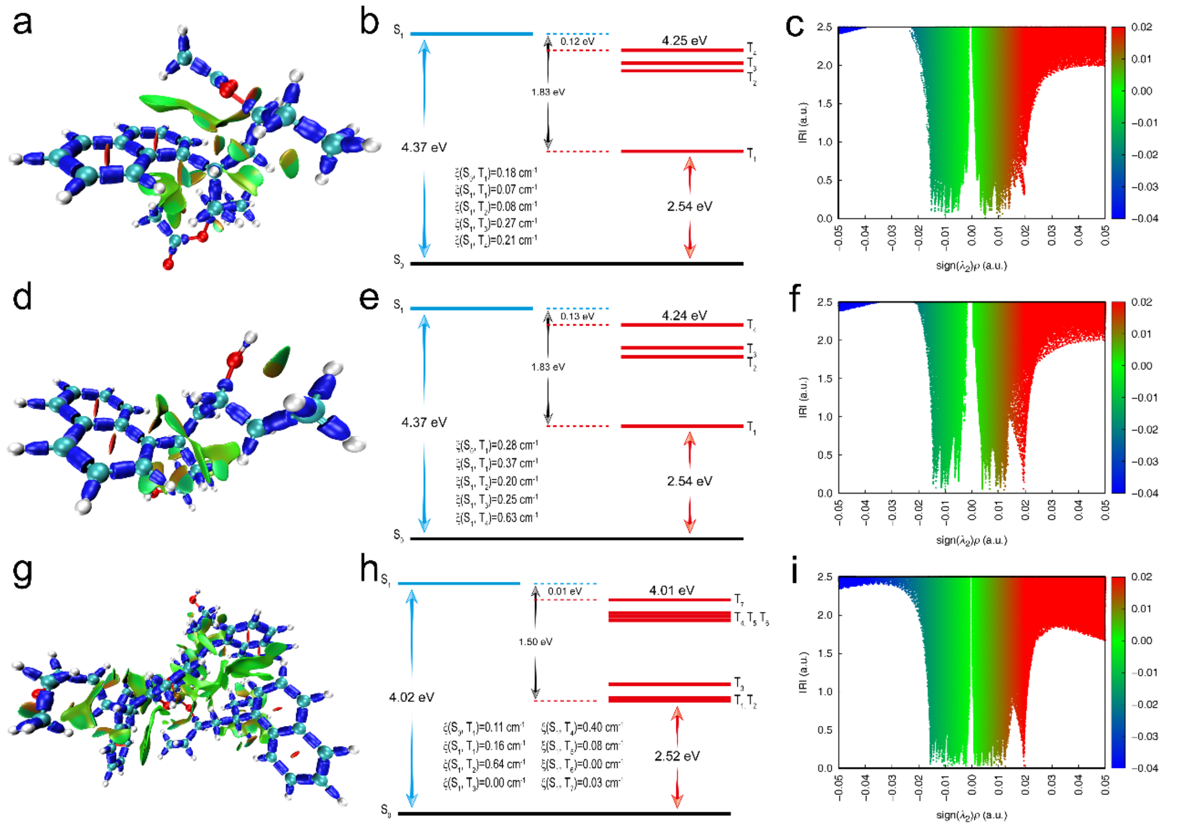

Supplementary Figure 40. Calculation studies. a-i 1VN: visualization of interactions in polymerization (**a**), alcoholysis (**d**), and cross-linked (**g**) structures; Theoretical calculation of the optimized simplified model: vertical excitation energy of polymerization (**b**), alcoholysis (**e**), and cross-linked (**h**) with boosted RTP performance: Vertical excitation energies and spin-orbit coupling constant (SOC); Distribution of different interactions via sign $(\lambda_2)\rho$ on IRI isosurfaces in polymerization (**c**), alcoholysis (**f**), and cross-linked (**i**). a.u.: atomic units.

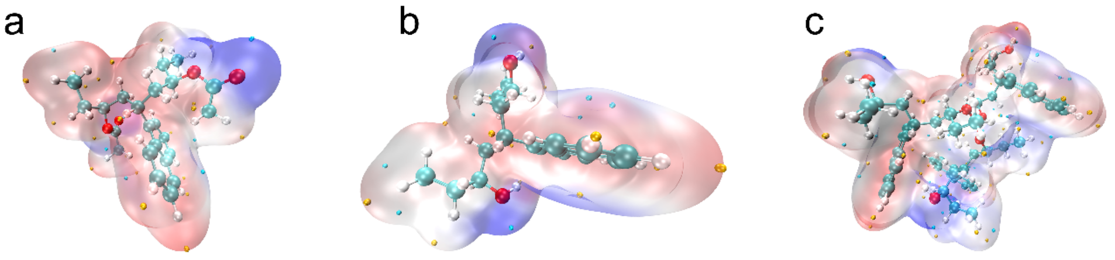

Supplementary Figure 41. Distribution of ESP. a-c Distribution of ESP in polymerization (**a**), alcoholysis (**b**), and cross-linked (**c**) structures. Negative potential (blue), positive potential (red), and neutral potential (white). The balls are the extreme point of regional potential (blue: minimum value; yellow: maximum value).

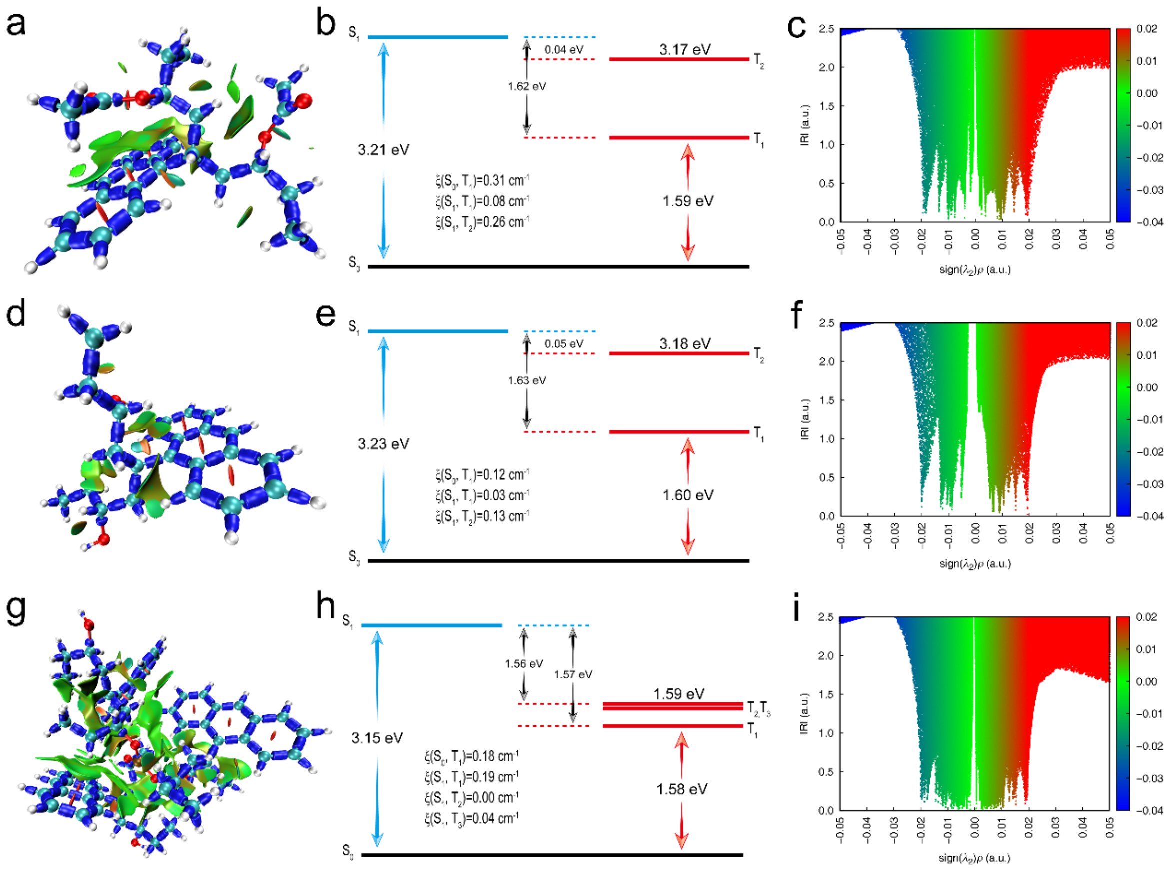

Supplementary Figure 42. Calculation studies. a-i 9VA: visualization of interactions in polymerization (**a**), alcoholysis (**d**), and cross-linked (**g**) structures; Theoretical calculation of the optimized simplified model: vertical excitation energy of polymerization (**b**), alcoholysis (**e**), and cross-linked (**h**) with boosted RTP performance: Vertical excitation energies and spin-orbit coupling constant (SOC); Distribution of different interactions via sign (λ_2)ρ on IRI isosurfaces in polymerization (**c**), alcoholysis (**f**), and cross-linked (**i**). a.u.: atomic units.

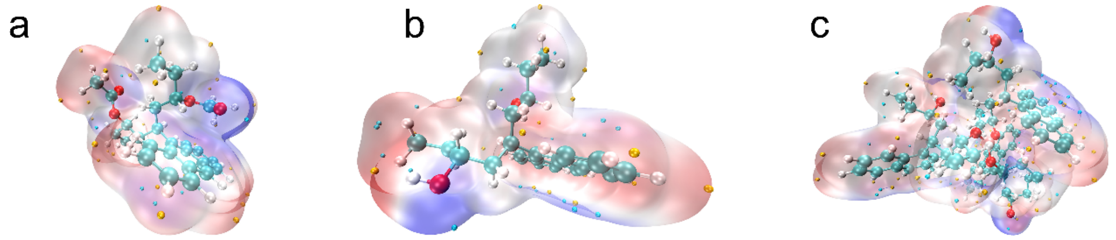

Supplementary Figure 43. Distribution of ESP. a-c Distribution of ESP in polymerization (**a**), alcoholysis (**b**), and cross-linked (**c**) structures. Negative potential (blue), positive potential (red), and neutral potential (white). The balls are the extreme point of regional potential (blue: minimum value; yellow: maximum value).

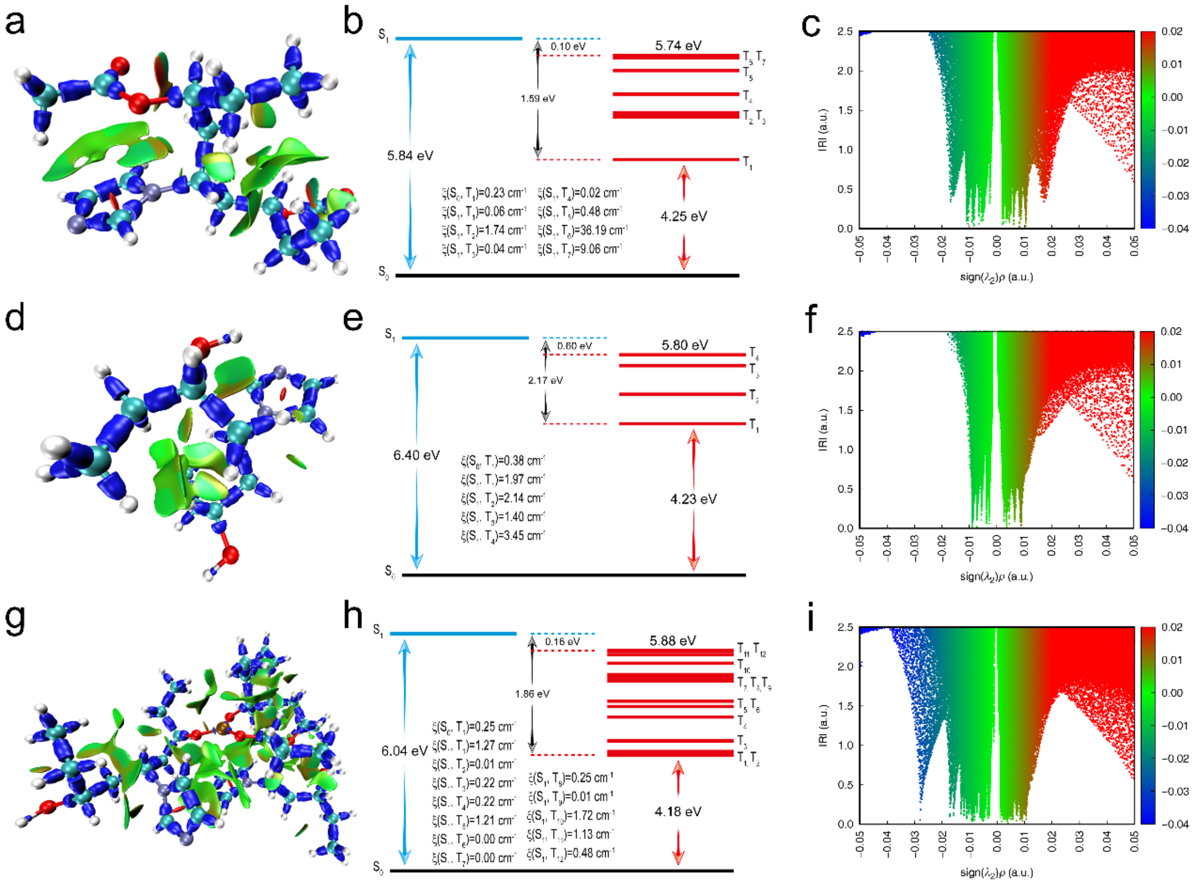

Supplementary Figure 44. Calculation studies. a-i MZ: visualization of interactions in polymerization (**a**), alcoholysis (**d**), and cross-linked (**g**) structures; Theoretical calculation of the optimized simplified model: vertical excitation energy of polymerization (**b**), alcoholysis (**e**), and cross-linked (**h**) with boosted RTP performance: Vertical excitation energies and spin-orbit coupling constant (SOC); Distribution of different interactions via sign $(\lambda_2)\rho$ on IRI isosurfaces in polymerization (**c**), alcoholysis (**f**), and cross-linked (**i**). a.u.: atomic units.

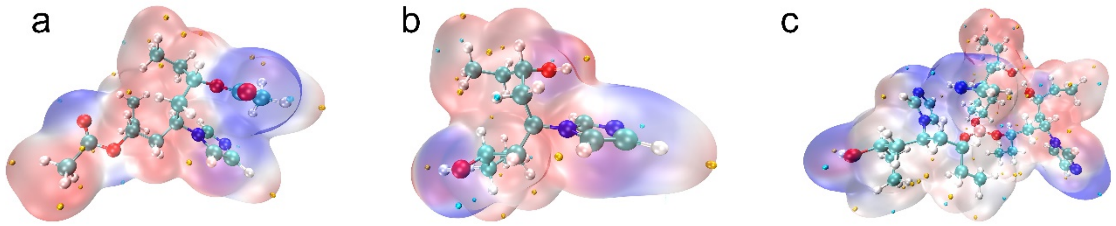

Supplementary Figure 45. Distribution of ESP. a-c Distribution of ESP in polymerization (**a**), alcoholysis (**b**), and cross-linked (**c**) structures. Negative potential (blue), positive potential (red), and neutral potential (white). The balls are the extreme point of regional potential (blue: minimum value; yellow: maximum value).

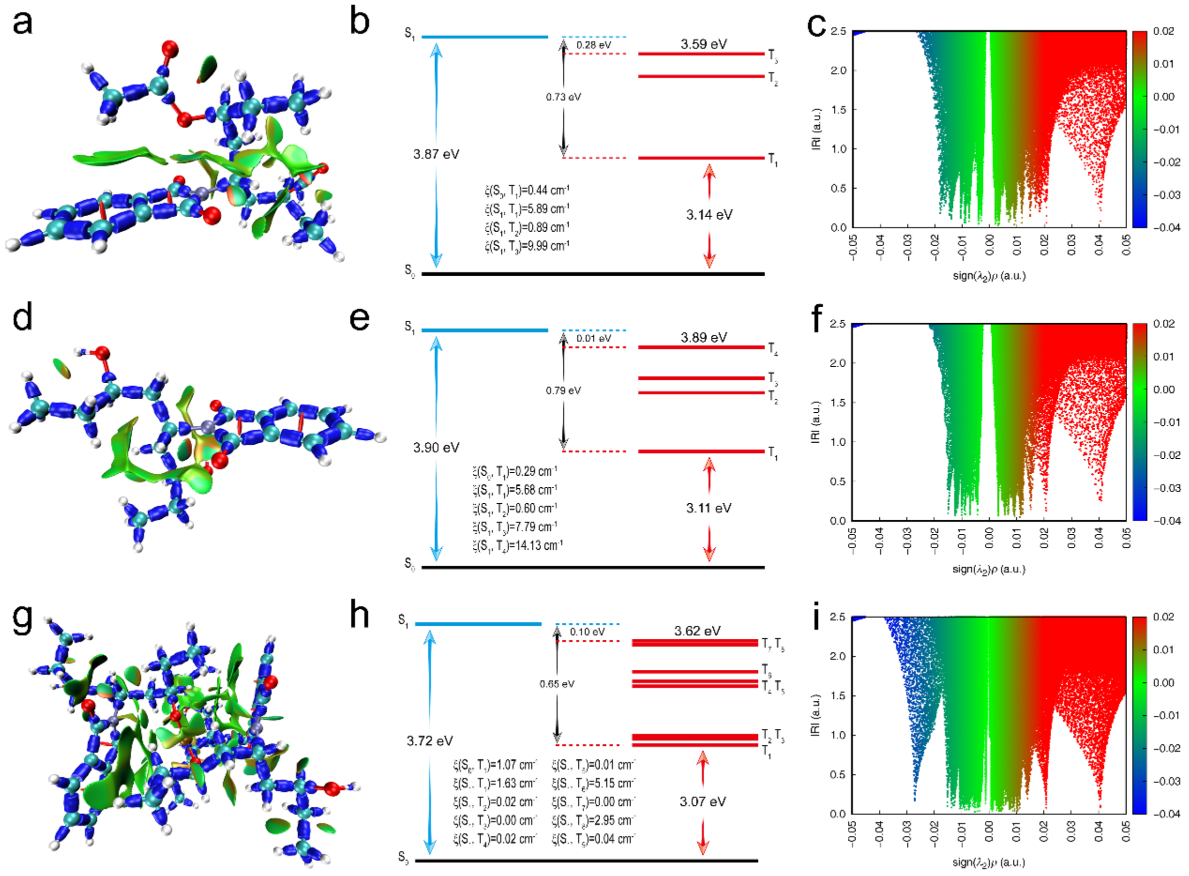

Supplementary Figure 46. Calculation studies. a-i NVP: visualization of interactions in polymerization (**a**), alcoholysis (**d**), and cross-linked (**g**) structures; Theoretical calculation of the optimized simplified model: vertical excitation energy of polymerization (**b**), alcoholysis (**e**), and cross-linked (**h**) with boosted RTP performance: Vertical excitation energies and spin-orbit coupling constant (SOC); Distribution of different interactions via sign (λ_2)ρ on IRI isosurfaces in polymerization (**c**), alcoholysis (**f**), and cross-linked (**i**). a.u.: atomic units.

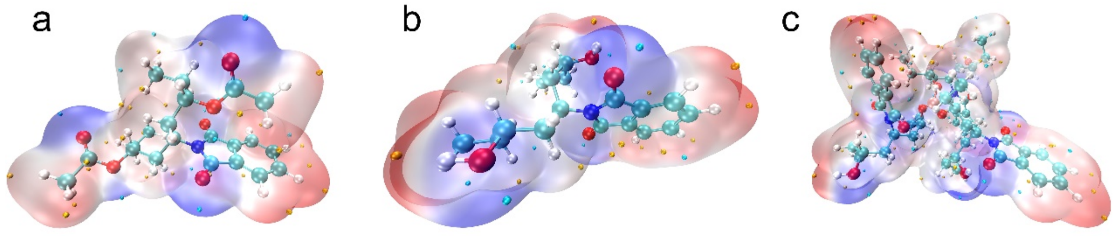

Supplementary Figure 47. Distribution of ESP. a-c Distribution of ESP in polymerization (**a**), alcoholysis (**b**), and cross-linked (**c**) structures. Negative potential (blue), positive potential (red), and neutral potential (white). The balls are the extreme point of regional potential (blue: minimum value; yellow: maximum value).

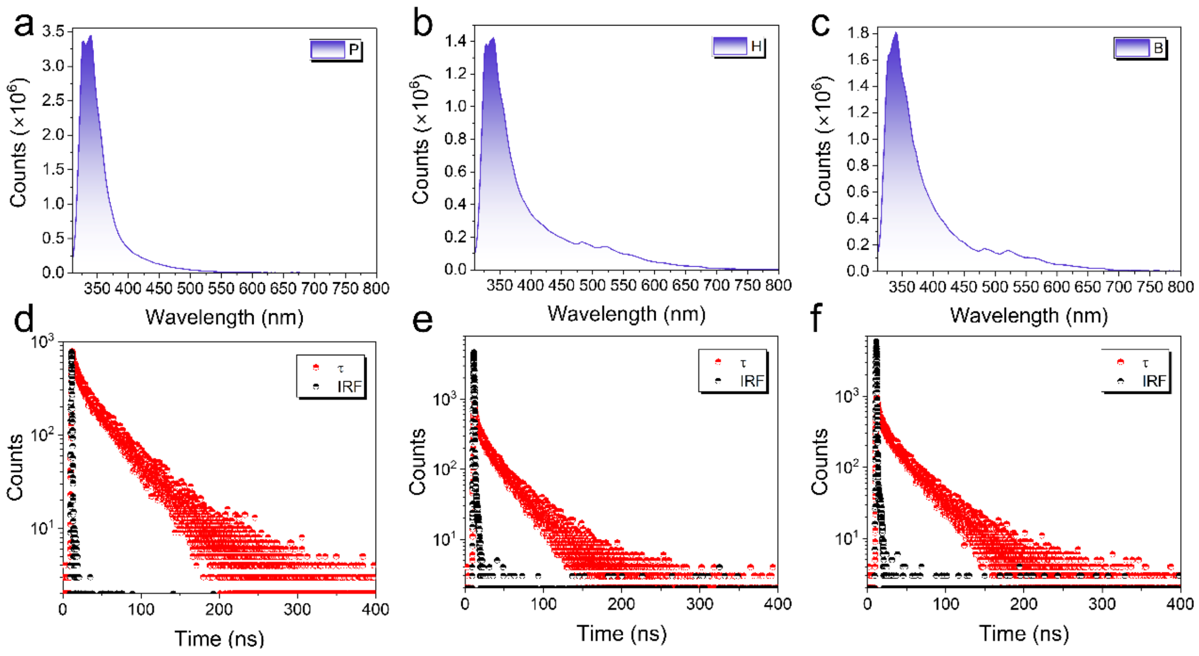

Supplementary Figure 48. Photoluminescence spectra and decay spectra. a-c Photoluminescence spectra of 1VN at λ_{ex} = 300 nm from P (**a**), H (**b**), and B (**c**). **d-f** Photoluminescence decay spectra with IRF at λ_{em} = 340 nm, 340 nm, and 340 nm for P (**d**), H (**e**), and B (**f**).

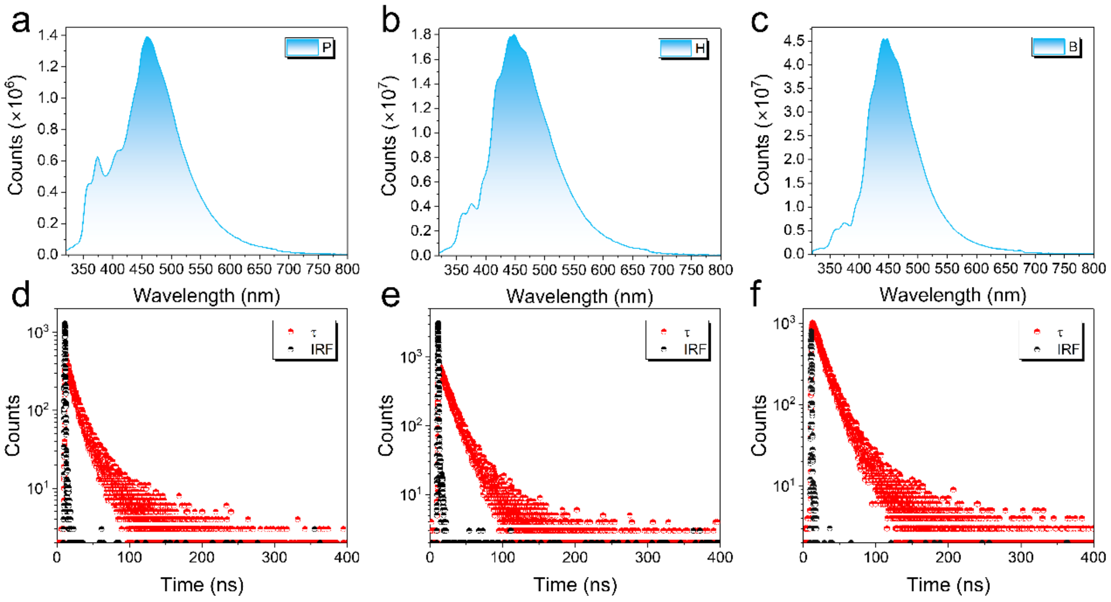

Supplementary Figure 49. Photoluminescence spectra and decay spectra. a-c Photoluminescence spectra of 9VA at λ_{ex} = 300 nm from P (**a**), H (**b**), and B (**c**). **d-f** Photoluminescence decay spectra with IRF at λ_{em} = 450 nm, 448 nm, and 448 nm for P (**d**), H (**e**), and B (**f**).

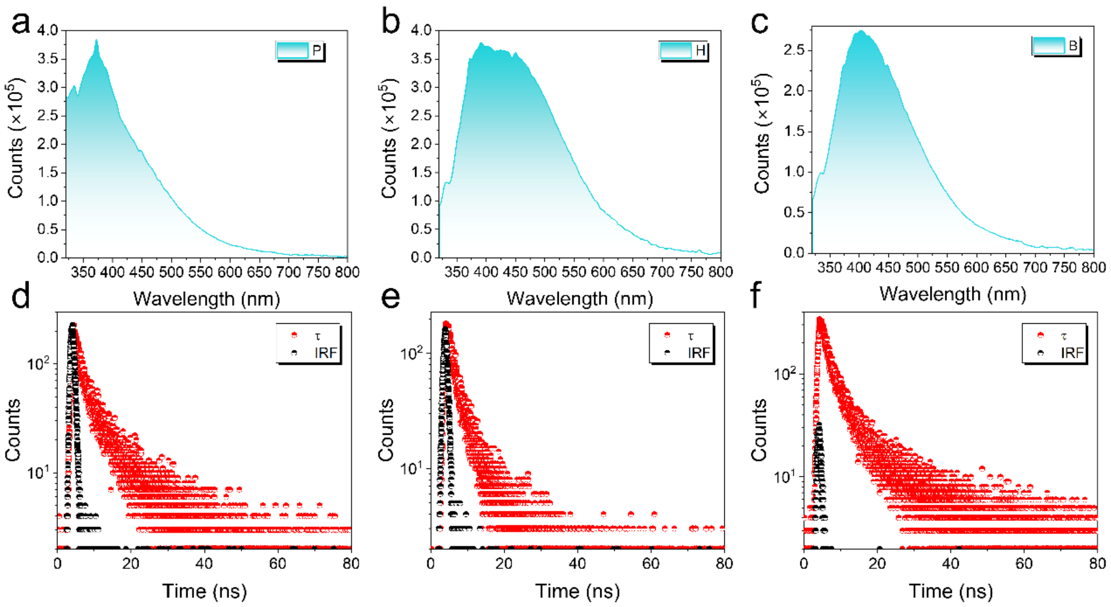

Supplementary Figure 50. Photoluminescence spectra and decay spectra. a-c Photoluminescence spectra of MZ at λ_{ex} = 300 nm from P (**a**), H (**b**), and B (**c**). **d-f** Photoluminescence decay spectra with IRF at λ_{em} = 372 nm, 392 nm, and 402 nm for P (**d**), H (**e**), and B (**f**).

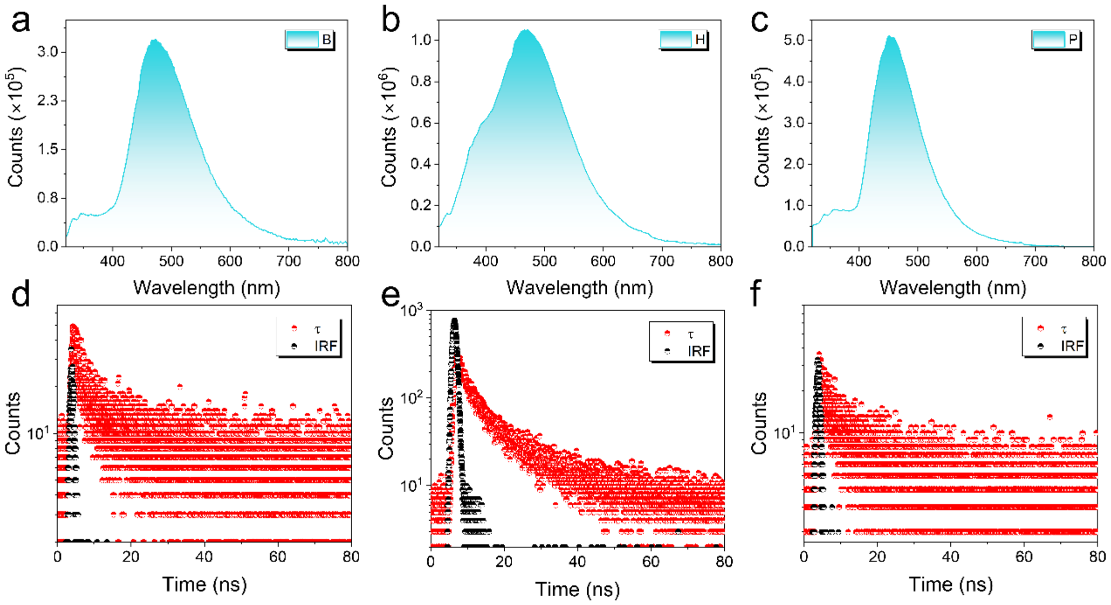

Supplementary Figure 51. Photoluminescence spectra and decay spectra. a-c Photoluminescence spectra of NVP at λ_{ex} = 300 nm from P (**a**), H (**b**), and B (**c**). **d-f** Photoluminescence decay spectra with IRF at λ_{em} = 452 nm, 466 nm, and 472 nm for P (**d**), H (**e**), and B (**f**).

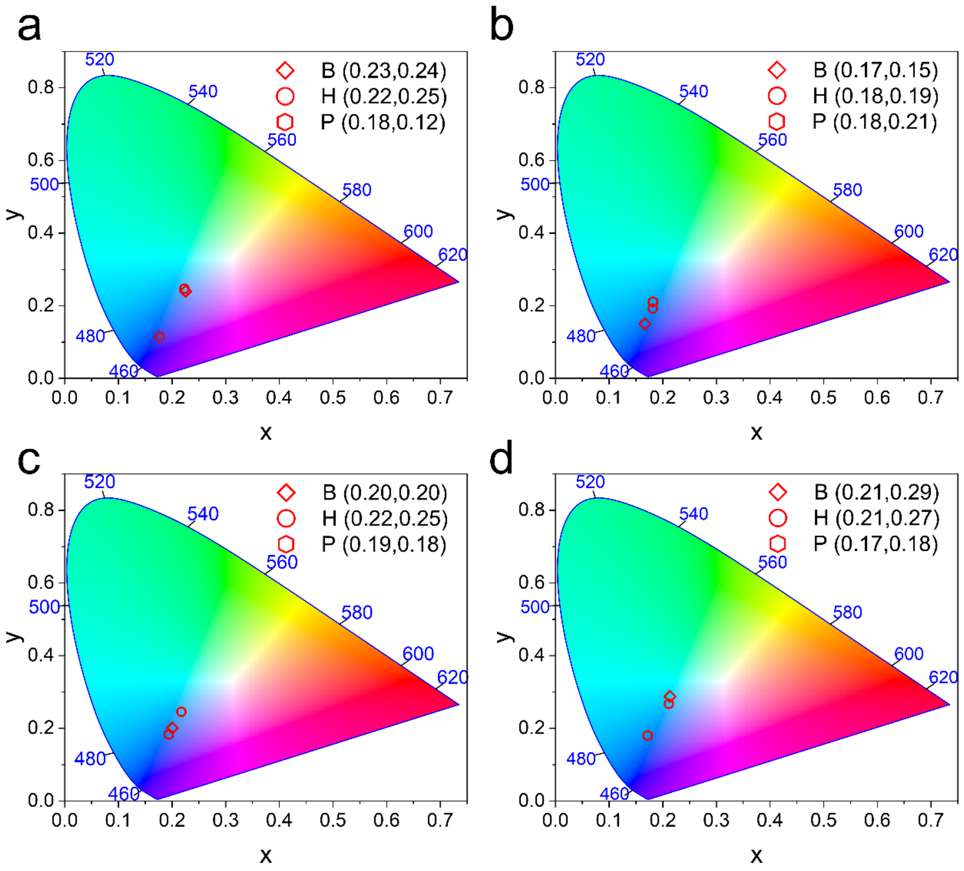

Supplementary Figure 52. CIE coordinates. a-d CIE coordinates from the photoluminescence spectra exhibiting a blue color.

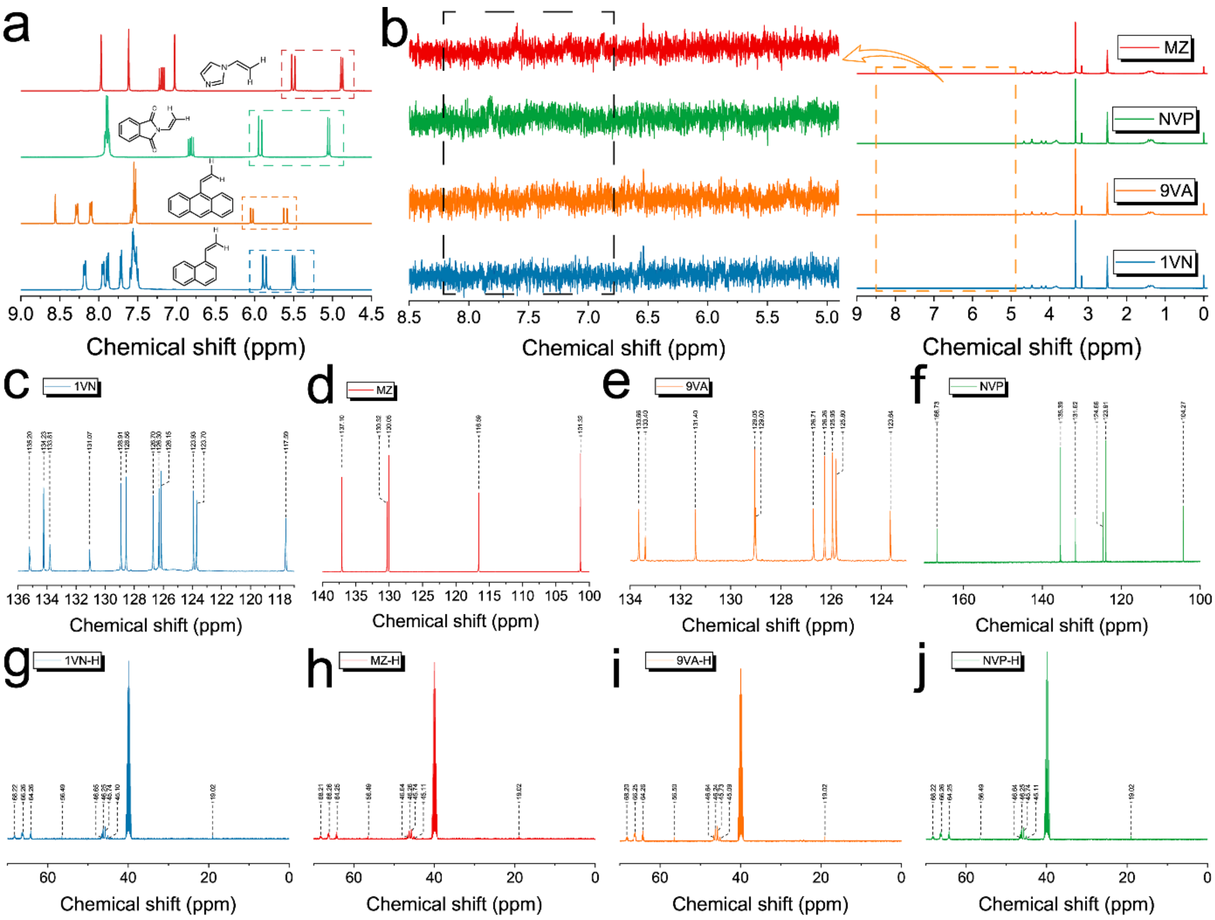

Supplementary Figure 53. ^{1}H and ^{13}C NMR spectra. a,b ^{1}H NMR of phosphors (**a**) and the copolymer after alcoholysis (**b**) obtained from DMSO-d_6. **c-j** ^{13}C NMR of different phosphors (**c-f**) and their copolymer after alcoholysis (**g-j**) obtained from DMSO-d_6. The signal of H from the vinyl group at 5.0-6.2 ppm in 1VN, 9VA, MZ, and NVP became nearly undetectable in copolymers upon converting to the alcohol hydroxyl at 4.22-4.67 ppm, while it additionally gathered aromatic ring H vibrations around 7.0-8.5 ppm (black dotted box).

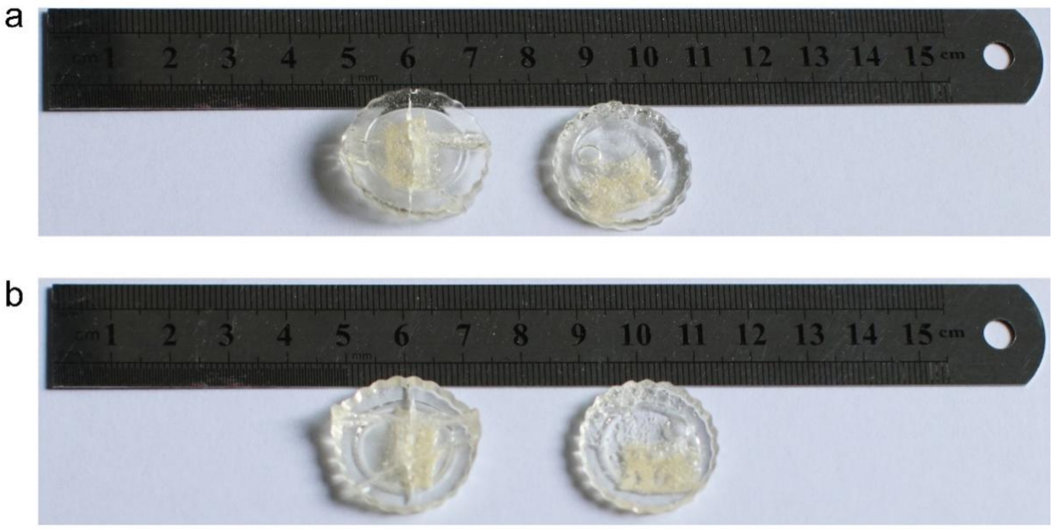

Supplementary Figure 54. Pictures of crack. a,b Pictures showing the sample with the crack (left) and the control group (right) of the back (**a**) and front (**b**) in the sunlight, as well as the shape of the crack.

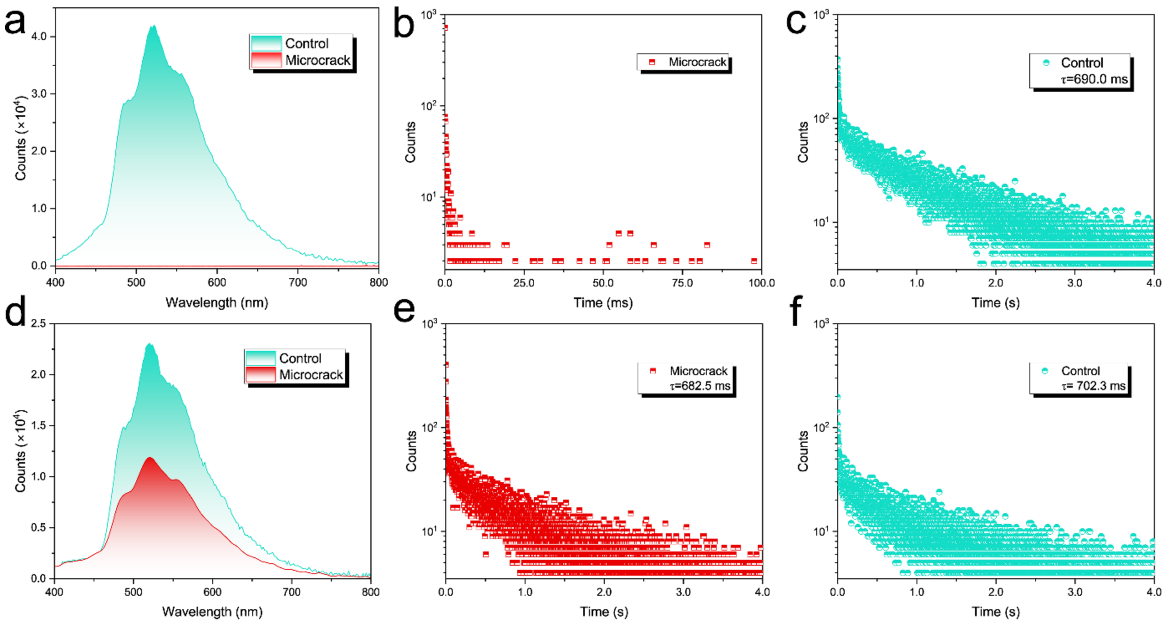

Supplementary Figure 55. Phosphorescence spectra as well as decay spectra and their lifetime. a-f Phosphorescence spectra of control and microcrack groups after fumigation (**a**) and the recovery (**d**); Decay spectra and their lifetime of control and microcrack groups after fumigation (**b, c**) and the recovery (**e, f**). The cracked sample was pretreated by fumigation, followed by cooling down to room temperature. The control parts were exposed to ambient conditions during the experiments for this period. These two groups of phosphorescence were compared under the same conditions.

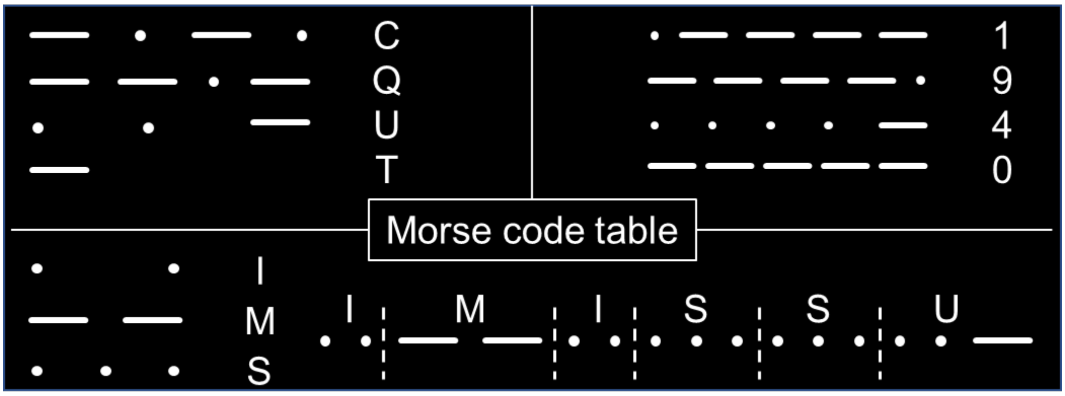

Supplementary Figure 56. Morse password decryption comparison table.

Supplementary Tables

Supplementary Table 1. Properties of copolymers with functionalized phosphors groups to develop ambient RTP materials from recent research.

Sample		Em/nm	τ/ms	Φ_P/%	Reference
PAA	PAA	488	31.7	4.5	12
	PAANa	478	124.9	7.6	12
	PBPh	485	1646.1	5.3	13
	R-PBNA	520	88.7	30.6	14
PAM	PH-1	490	1463	10.1	15
	PAM-N1	494	1534.6	12.0	16
	BAA	477	351	N/A	17
PSS	PSSLi	550	1308	N/A	18
	PSS	496	1007	N/A	19
PVP	PVP-S	524	578.4	6.4	20

Note: PAA: polyacrylic acid; PAM: polyacrylamide; PSS: polystyrene sulfonic acid; PVP: polyvinyl pyridine, Em: emission wavelength, τ: phosphorescence lifetime, Φ_P: phosphorescence quantum yields. N/A: no recorded data found.

Supplementary Table 2. Naming of series 1 to 4 and different phosphorescence units.

VAc : Phosphor	2VN				1VN	9VA	MZ	NVP
	300:1	500:1	700:1	1000:1				
**Polymerization (P)**	P1	P2	P3	P4	1VN-P	9VA-P	MZ-P	NVP-P
**Alcoholysis (H)**	H1	H2	H3	H4	1VN-H	9VA-H	MZ-H	NVP-H
**Cross-linking (B)**	B1	B2	B3	B4	1VN-B	9VA-B	MZ-B	NVP-B

Note: VAc : Phosphor means molar ratio.

Supplementary Table 3. Photophysical parameters of 2VN with different feed radios of polymerization (P), alcoholysis (H), and crosslinked (B) under ambient conditions.

	Fluorescence				Phosphorescence				
Systems	Em/nm	τ_F/ns	Φ_F/%	k_{isc}/s^{-1}	Em/nm	τ_P/ms	Φ_P/%	k_r^P/s^{-1}	k_{nr}^P/s^{-1}
B1	338	41.2	18.73	1.0×10^{6}	520	1197.5	4.29	3.6×10^{-2}	8.0×10^{-1}
H1	340	35.4	19.97	6.2×10^{5}	520	721.2	2.19	3.0×10^{-2}	1.4×10^{0}
P1	340	45.8	45.57	6.6×10^{3}	558	0.3	0.03	1.0×10^{0}	3.3×10^{3}
B2	336	34.8	10.37	1.6×10^{6}	520	1230.4	5.64	4.6×10^{-2}	7.7×10^{-1}
H2	338	30.9	10.31	1.0×10^{6}	520	753.9	3.11	4.1×10^{-2}	1.3×10^{0}
P2	336	36.6	44.80	3.0×10^{4}	522	1.4	0.11	7.9×10^{-1}	7.1×10^{2}
B3	336	42.0	21.37	2.4×10^{6}	520	1263.6	10.17	8.0×10^{-2}	7.1×10^{-1}
H3	338	38.8	10.12	6.4×10^{5}	520	761.8	2.50	3.3×10^{-2}	1.3×10^{0}
P3	338	50.8	43.27	1.2×10^{4}	518	4.7	0.06	1.3×10^{-1}	2.1×10^{2}
B4	340	40.2	11.12	1.6×10^{6}	520	1231.9	6.52	5.3×10^{-2}	7.6×10^{-1}
H4	340	35.5	13.27	9.8×10^{5}	522	720.4	3.47	4.8×10^{-2}	1.3×10^{0}
P4	340	43.2	44.09	2.8×10^{4}	508	4.7	0.12	2.6×10^{-1}	2.1×10^{2}

Supplementary Table 4. Molecular weights by GPC for the alcoholysis systems from 1VN, 2VN, 9VA, MZ, and NVP with water as the mobile phase.

Systems		Mn (Daltons)	Mw (Daltons)	Mp (Daltons)	Mz (Daltons)	Mz+1 (Daltons)	PDI	Mz/Mw	Mz+1/Mw
2VN	H1	26549	50173	47547	77447	106971	1.89	1.54	2.13
	H2	23516	45143	40851	71652	102457	1.92	1.59	2.27
	H3	24183	44026	41056	67162	92457	1.82	1.53	2.10
	H4	25471	46278	43508	70394	96327	1.82	1.52	2.08
1VN-H		33029	63146	57810	101830	146714	1.91	1.61	2.32
9VA-H		29228	58191	51240	96671	140240	1.99	1.66	2.41
MZ-H		31120	61127	55068	101113	149582	1.96	1.65	2.44
NVP-H		31591	62928	57855	103105	149698	1.99	1.64	2.38

Note: Considering the solubility after cross-linking, the sample after alcoholysis was selected to characterize the molecular weight.

Supplementary Table 5. Photophysical parameters of polymerization (P), alcoholysis (H), and crosslinked (B) from different phosphors under ambient conditions.

Fluorescence					Phosphorescence				
systems	Em/nm	τ_F/ns	Φ_F/%	k_{isc}/s^{-1}	Em/nm	τ_P/ms	Φ_P/%	k_r^P/s^{-1}	k_{nr}^P/s^{-1}
**1VN**									
B	340	38.0	11.20	1.8×10^{6}	524	1247.1	6.72	5.4×10^{-2}	7.5×10^{-1}
H	340	31.4	8.46	1.4×10^{6}	524	1028.8	4.33	4.2×10^{-2}	9.3×10^{-1}
P	340	42.0	31.20	1.9×10^{4}	404	7.3	0.08	1.1×10^{-1}	1.4×10^{2}
**9VA**									
B	448	18.3	31.00	6.3×10^{5}	558	256.5	1.15	4.5×10^{-2}	3.9×10^{0}
H	448	15.1	17.24	4.0×10^{5}	570	95.9	0.60	6.3×10^{-2}	1.0×10^{1}
P	450	22.0	11.61	0	566	14.3 μs	N/A	0	7.0×10^{4}
**NVP**									
B	472	7.5	11.31	2.1×10^{7}	482	338.3	16.04	4.7×10^{-1}	2.5×10^{0}
H	466	2.7	7.42	1.4×10^{7}	490	262.3	3.68	1.4×10^{-1}	3.7×10^{0}
P	452	8.3	17.47	1.3×10^{6}	480	24.2	1.05	4.3×10^{-1}	4.1×10^{1}
**MZ**									
B	402	4.2	7.18	2.8×10^{6}	486	499.1	1.19	2.4×10^{-2}	2.0×10^{0}
H	392	3.7	3.44	1.4×10^{6}	492	281.3	0.52	1.8×10^{-2}	3.5×10^{0}
P	372	5.4	4.38	1.5×10^{5}	510	1.2	0.08	6.7×10^{-1}	8.3×10^{2}

Note: Phosphorescence quantum yield of 9VA-P was too weak to be detected and thus represented by N/A. When calculating k_{isc} and k_r^P, Φ_P is treated as 0.

Supplementary References

1. Feng, H. et al. Tuning molecular emission of organic emitters from fluorescence to phosphorescence through push-pull electronic effects. *Nat. Commun.* **11**, 2617 (2020).
2. Tao, Y. et al. Resonance-activated spin-flipping for efficient organic ultralong room-temperature phosphorescence. *Adv. Mater.* **30**, 1803856 (2018).
3. Zhang, Y. et al. Ultraviolet irradiation-responsive dynamic ultralong organic phosphorescence in polymeric systems. *Nat. Commun.* **12**, 2297 (2021).
4. Wang, X. et al. Organic phosphors with bright triplet excitons for efficient X-ray-excited luminescence. *Nat. Photon*. **15**, 187–192 (2021).
5. Zhu, T. et al. Clustering and halogen effects enabled red/near-infrared room temperature phosphorescence from aliphatic cyclic imides. *Nat. Commun.* **13**, 2658 (2022).
6. Zhou, W. L. et al. Ultralong purely organic aqueous phosphorescence supramolecular polymer for targeted tumor cell imaging. *Nat. Commun.* **11**, 4655 (2020).
7. Neese, F., Wennmohs, F., Becker, U. & Riplinger, C. The ORCA quantum chemistry program package. *J. Chem. Phys.* **152**, 224108 (2020).
8. Manzetti, S. & Lu, T. The geometry and electronic structure of aristolochic acid: possible implications for a frozen resonance. *J. Phys. Org. Chem.* **26**, 473–483 (2013).
9. Zhang, J. & Lu, T. Efficient evaluation of electrostatic potential with computerized optimized code. *Phys. Chem. Chem. Phys.* **23**, 20323–20328 (2021).
10. Lu, T. & Chen, F. Multiwfn: A multifunctional wavefunction analyzer. *J. Comput. Chem.* **33**, 580–592 (2012).
11. Humphrey, W., Dalke, A. & Schulten, K. VMD: visual molecular dynamics. *J. Molec. Graph.* **14**, 33–38 (1996).
12. Zhou, Q. et al. Emission mechanism understanding and tunable persistent room temperature phosphorescence of amorphous nonaromatic polymers. *Mater. Chem. Front.* **3**, 257–264 (2019).
13. Gan, N. et al. Organic phosphorescent scintillation from copolymers by X-ray irradiation. *Nat. Commun.* **13**, 3995 (2022).
14. Gu, L. et al. Circularly polarized organic room temperature phosphorescence from

amorphous copolymers. *J. Am. Chem. Soc.* **143**, 18527–18535 (2021).

15. Wang, S., Wu, D., Yang, S., Lin, Z. & Ling, Q. Regulation of clusterization-triggered phosphorescence from a non-conjugated amorphous polymer: a platform for colorful afterglow. *Mater. Chem. Front.* **4**, 1198–1205 (2020).
16. Zhang, Z. Y. et al. A synergistic enhancement strategy for realizing ultralong and efficient room-temperature phosphorescence. *Angew. Chem. Int. Ed.* **59**, 18748–18754 (2020).
17. Wu, Q. et al. Self-Healing Amorphous Polymers with Room-Temperature Phosphorescence Enabled by Boron-Based Dative Bonds. *ACS Appl. Polym. Mater.* **2**, 699–705 (2020).
18. Ogoshi, T. et al. Ultralong room-temperature phosphorescence from amorphous polymer poly(styrene sulfonic acid) in air in the dry solid state. *Adv. Funct. Mater.* **28**, 1707369 (2018).
19. Cai, S. et al. Enabling long-lived organic room temperature phosphorescence in polymers by subunit interlocking. *Nat. Commun.* **10**, 4247 (2019).
20. Wang, H. et al. Amorphous ionic polymers with color-tunable ultralong organic phosphorescence. *Angew. Chem. Int. Ed.* **58**, 18776–18782 (2019).
