# Supplementary material for: Stepwise taming of triplet excitons via multiple confinements in intrinsic polymers for long-lived room-temperature phosphorescence
Source: Nat Commun. 2023 Nov 9;14:7252. doi: 10.1038/s41467-023-43133-1 (PMC10636106; doi:10.1038/s41467-023-43133-1)
Supplement: Supplementary file 3 — Description of Additional Supplementary Files [file 41467_2023_43133_MOESM3_ESM.pdf]

Description of Additional Supplementary Files

File Name: Supplementary Movie 1
Description: The afterglow of P3, H3, and B3 excited by a 254 nm UV lamp.

File Name: Supplementary Movie 2
Description: The afterglow of P1, H1, and B1 excited by a 254 nm UV lamp.

File Name: Supplementary Movie 3
Description: The afterglow of P2, H2, and B2 excited by a 254 nm UV lamp.

File Name: Supplementary Movie 4
Description: The afterglow of P4, H4, and B4 excited by a 254 nm UV lamp.

File Name: Supplementary Movie 5
Description: The afterglow of 1VN with P, H, and B excited by a 254 nm UV lamp.

File Name: Supplementary Movie 6
Description: The afterglow of 9VA with P, H, and B excited by a 254 nm UV lamp.

File Name: Supplementary Movie 7
Description: The afterglow of MZ with P, H, and B excited by a 254 nm UV lamp.

File Name: Supplementary Movie 8
Description: The afterglow of NVP with P, H, and B excited by a 254 nm UV lamp.

File Name: Supplementary Movie 9
Description: The comparison of phosphorescence in microcrack detection and recovery after the excitation of 254 nm UV lamp.

File Name: Supplementary Movie 10
Description: The phosphorescence combined with Morse code after the excitation of 254 nm UV lamp.

File Name: Supplementary Data 1

Description: Atomic coordinates of optimized computational models for 1VN-B, 1VN-H, 1VN-P, 2VN-B, 2VN-H, 2VN-P, 9VA-B, 9VA-H, 9VA-P, MZ-B, MZ-H, MZ-P, NVP-B, NVP-H, and NVP-P.
